# Supplementary material for: COVID-19 and systemic lupus erythematosus genetics: A balance between autoimmune disease risk and protection against infection
Source: PLoS Genet. 2022 Nov 3;18(11):e1010253. doi: 10.1371/journal.pgen.1010253 (PMC9632821; doi:10.1371/journal.pgen.1010253)
Supplement: S1 Text — (DOCX) [file pgen.1010253.s001.docx]

**COVID-19 and systemic lupus erythematosus genetics: a balance between autoimmune disease risk and protection against infection**

**Yuxuan Wang^1^, Suri Guga^1^, Kejia Wu^1^, Zoe Khaw^1^, Konstantinos Tzoumkas^1^, Phil Tombleson^2^, Mary E. Comeau^3^, Carl D. Langefeld^3^, Deborah S. Cunninghame Graham^1^, David L. Morris*^,1,¶^, Timothy J. Vyse^1,¶^**

**^1^ Department of Medical & Molecular Genetics, King’s College London, London, UK.**

**^2^ NIHR GSTFT/KCL Biomedical Research Centre, London, UK.**

**^3^ Department of Biostatistics and Data Science, Wake Forest School of Medicine, Winston-Salem, North Carolina, United States of America**

**^¶^ These authors contributed equally.**

***** [**david.l.morris@kcl.ac.uk**](mailto:david.l.morris@kcl.ac.uk)

**Supplementary Results**

***TYK2* associations. Extended results.**

**Table F** displays the association results from the analyses in the main paper and is identical to **Table 2** but with additional information. **Table F** includes the summary statistics for SLE for the SNPs reported as associated with severe COVID-19 and vice versa. This table also includes the 3^rd^ associated variant rs74908652 from stepwise regression in the SLE data. rs74908652 is in LD (r^2^ = 0.44) with the second signal in the analyses of the individual data (rs12720356, see **Table I**).

Stepwise regression in the SLE individual level data using SNPTEST resulted in four independently associated SNPs (see **Table I**). The most associated with SLE is a missense variant rs34536443 (P1104A) that is in high LD (r^2^=0.88) with rs74956615. Conditional on rs34536443 the next two most associated SNPs are in weak LD (r^2^=0.24 and r^2^=0.35, respectively) with rs11085727: one is missense (rs12720356, I684S) and the other is non-coding (rs280497). The 4^th^ associated SNP rs12720358 is a relatively rare non-coding variant. We replicated the lead SLE signal tagged by rs34536443 in the Hispanic Immunochip data (see **Table J**), rs34536443 was the most associated SNP in these data (p = 1.28 x 10^-06^, MAF_cases_ = 0.006, MAF_controls_ = 0.021) and was in LD (r^2^ = 0.73) with rs74956615. We did not find significant evidence for additional signals in the Hispanic data. While we found no evidence for association in the African-American (AA) data, the estimated direction of effects for all SNPs in **Tables 2 and I in S1 Text** agreed with the EUR data (see **Table J**). We also found this to be true for the Hispanic data.

There are multiple studies showing evidence of coding variation associated with SLE and other AID, and there is some understanding of how these are functionally related to disease. A study[1] of several AID, including SLE found evidence for association with rs12720356 which is missense and is an associated variant when we run stepwise regression on the individual level SLE data (see methods and **Table I**). Another study in rheumatoid arthritis (RA) and SLE[2] found evidence of association with this coding variant and also with rs35018800, a rare (MAF = 0.003) variant that we do find associated with SLE in our data at genome wide level when conditioning on the top four associated SLE variants (**Table I**). The open question is whether these coding variants act independently of the variant rs11085727, that we find tags the colocalized eQTL signal for *TYK2,* and a missense variant rs2304256 that is in strong LD (r^2^ = 0.98 in SLE data) with rs11085727, and in the same credibility set for SLE, that is a significant eQTL for *TYK2* (Effect Allele = A, BETA = 0.249, Bonferroni P = 8.15 x 10^-160^, from eQTLGen[3] (whole blood)). This eQTL also replicates in naïve monocytes[4]. The RA and SLE[2] study suggested, through haplotype analysis, that the rs2304256 association could be driven by imperfect LD with the three missense variants rs34536443, rs12720356 and rs35018800. A similar conclusion was reached in a study of systemic sclerosis patients[5] and psoriasis[6]. Another fine-mapping study of RA and type 1 diabetes (T1D)[7] identified rs34536443, rs12720356 and rs35018800 as associated, but not rs2304256. These results suggest that the *TYK2* regulatory variants may only be associated with disease through their LD with the coding variants. However, along with the evidence we find of independent association in the results presented in **Tables 2 and I in S1 Text**, our haplotype analysis using the largest individual SLE data (Immunochip) did find evidence (p = 0.01) for an independent effect for rs11085727 and rs2304256, with the [TA] haplotype having a protective effect, over and above haplotypes tagged by the other SNPs in **Tables 2 and I in S1 Text**. We did not see significant evidence for this in our other EUR GWAS data, but the direction of effect was the same.

Data from East Asian populations suggest that there may also be rare coding and common regulatory variants associated with SLE at *TYK2*. The missense variants (rs34536443, rs12720356 and rs35018800) that we find associated with SLE in Europeans are rarer in the East Asian populations (1000 Genomes frequencies: 0.03, 0.09, 0.003 versus 0, 0, 0). rs11085727/ rs2304256 are more common (1000 Genomes frequencies: 0.27, 0.26 versus 0.56, 0.52). One study in Chinese^8^ failed to find statistically significant association at *TYK2*. In contrast, a previous study found strong evidence for the missense SNP rs2304256 association^9^, and in agreement with our EUR data the C allele was reported as risk for SLE. Another recent study in East Asian populations^10^ found one strong association with a missense SNP rs55882956 (Arg703Trp; effect allele = A[G], freq(A) = 0.03), which is very rare in Europeans (freq (A) = 0.00) and not associated in our data.

**Epigenetic modification and chromatin looping at *TYK2***

There are active regions next to the missense SNP (rs34536443) in signal A (**Figs O-P**(zoomed in)). This is observed in several cell lines using data from ENCODE. Signal-A is also observed to loop in 3D space to the promotor of *PDE4A* (**Fig R**)**.**

The signal-B SNPs rs2302456 and rs34725611 are in an active region showing signals of H3K4Me1 on GM12878 Human B lymphocyte cell line (**Fig O** and zoomed in at **Fig Q**).

| **SLE GWAS datasets** | | | | | |
| --- | --- | --- | --- | --- | --- |
| **Dataset** | **Population** | **Phenotype** | **Case** | **Control** | **Citation** |
| SLEGEN cohort | EUR | SLE vs. European population controls | 533 | 2,543 | [8] |
| Genentech cohort | EUR | SLE vs. European population controls | 1,165 | 2,107 | [9] |
| SLE main cohort | EUR | SLE vs. European population controls | 4,036 | 6,959 | [10] |
| Immunochip_EUR | EUR | SLE vs. European population controls | 3,568 | 11,245 | [11] |
| **COVID-19 GWAS datasets** | | | | | |
| **Dataset** | **Population** | **Phenotype** | **Case** | **Control** | **Citation** |
| GenOIMICC_EUR | EUR | critically ill patients with COVID-19 vs. European UK Biobank controls | 1,676 | 8,380 | [12] |
| COVID19-hg A2_ALL_leave_23andme | All | very severe respiratory confirmed COVID-19 vs. population | 8,779 (6,956 EUR) | 1,001,875 (673,564 EUR) | [13] |
| **Table A. Sample sizes and data sources of SLE/COVID-19 GWAS data used in the study.** | | | | | |

| **Table B. Top 10 genome partitions from ρ-HESS analysis** | | | | | | | |
| --- | --- | --- | --- | --- | --- | --- | --- |
| chr | start (bp) | end (bp) | Z score | p-value | N_SNP_ | nearest SLE  risk loci | nearest COVID-19 risk loci |
| 19 | 9,238,393 | 11,284,028 | -3.88 | 1.06 x 10^-04^ | 2,544 | *TYK2, PDE4A, ICAM3,*  *KEAP1, ILF3[10, 11, 14-17]* | *TYK2, CDC37, RAVER1,*  *ZGLP1, ICAM1[12, 13, 18, 19]* |
| 6 | 31,571,218 | 32,682,664 | -2.42 | 1.53 x 10^-02^ | 495 | *HLA-DRB1, HLA-DQA1,AIF1, UQCRHP1, CSNK2B, MSH5, MSH5-SAPCD1, HSPA1B, HSPA1A, SNHG32, NEU1, SLC44A4, C2, CFB, C4B, CYP21A1P, CYP21A2, C4B-AS1, TNXB, PRRT1, FKBPL, NOTCH4, TSBP1-AS1, TSBP1, BTNL2, HLA-DRA, HLA-DRB9, HLA-DRB5, HLA-DQB1, MTCO3P1, HLA-DQB3[9-11, 15, 20-29]* | *HLA*-*DRB1*, *HLA*-*DQA1[18]* |
| 2 | 146,445,570 | 147,277,162 | -2.23 | 2.59 x 10^-02^ | 502 | *ARHGAP15*[30] | *LRP1B*, *GALNT13[19]* |
| 2 | 198,078,110 | 199,311,125 | 2.22 | 2.66 x 10^-02^ | 1,101 | *PLCL1[11, 20, 30]* | *ACER2P1, ZDBF2[19]* |
| 6 | 137,614,218 | 138,822,629 | 2.08 | 3.75 x 10^-02^ | 410 | *BTF3L4P3, LINC02539,*  *WAKMAR2,* *TNFAIP3,*  *LINC02528[10, 11, 14-16, 21, 25, 27, 28, 31-34]* | *NKAIN2[19]* |
| 1 | 221,858,231 | 222,230,220 | 2.06 | 3.96 x 10^-02^ | 462 | *TARBP1, LINC01354[35]* | *SLC30A10[19]* |
| 8 | 11,278,998 | 13,491,775 | -1.84 | 6.60 x 10^-02^ | 266 | *BLK, FAM167A,*  *LINC00208, GATA4[9-11, 14-16, 22, 23, 26-28, 31-34, 36-38]* | *KBTBD11[19]* |
| 7 | 42,001,811 | 43,159,074 | 1.82 | 6.86 x 10^-02^ | 2,027 | *SPATA48[28]* | *TOMM7[19]* |
| 19 | 36,469,295 | 37,527,033 | 1.79 | 7.35 x 10^-02^ | 1,506 | *ANKRD27[30]* | *FUT2[18]* |
| 10 | 60,289,987 | 61,891,409 | -1.73 | 8.32 x 10^-02^ | 2,165 | *ARID5B[10, 15, 20, 25]* | *CHST3[39]* |
| Heritability estimation from summary statistics (HESS) top 10 results of SLE meta-analysis data (3 EUR GWAS + Immunochip) vs. COVID-19 genOMMIC_EUR data. There were 7 negatively correlated genome partitions and 13 positively correlated genome partitions among the top 20. | | | | | | | |

| ***IRF8* associations with SLE** | | | | | | | | | | | |
| --- | --- | --- | --- | --- | --- | --- | --- | --- | --- | --- | --- |
| **Signal number** | **SNP** | **position** | **A2** | **A1** | **freq(A1)** | **BETA** | **SE** | **P** | **bJ** | **bJ_se** | **pJ** |
| Signal-A | rs9938016 | 16:86002524 | C | T | 0.205 | -0.22 | 0.02 | 3.60 x 10^-20^ | -0.21 | 0.02 | 1.29 x 10^-18^ |
| Signal-B | rs2970090 | 16:85967891 | G | A | 0.419 | 0.14 | 0.02 | 2.49 x 10^-13^ | 0.13 | 0.02 | 8.57 x 10^-12^ |
| ***IRF8* associations with severe COVID-19** | | | | | | | | | | | |
| **Signal number** | **SNP** | **position** | **A2** | **A1** | **freq(A1)** | **BETA** | **SE** | **P** | **bJ** | **bJ_se** | **pJ** |
| Signal-A | rs17445836 | 16:86017663 | G | A | 0.233 | 0.07 | 0.03 | 7.11 x 10^-03^ | 0.07 | 0.03 | 7.11 x 10^-03^ |
| **Table C. *IRF8* association results for a) SLE and b) severe COVID-19 data.** Independently associated SNPs in SLE and severe COVID-19 are displayed. * bJ, bJ_se, pJ: effect size, standard error and p-value from a joint analysis (multiple regression) of all the selected SNPs (results conditional on all other SNPs if selected from stepwise regression). ^ϯ^ rs17445836 is in high LD with rs9938016 (r^2^ = 0.71). | | | | | | | | | | | |

| ***TNFSF4* associations with SLE** | | | | | | | | | | | |
| --- | --- | --- | --- | --- | --- | --- | --- | --- | --- | --- | --- |
| **Signal number** | **SNP** | **position** | **A2** | **A1** | **freq(A1)** | **BETA** | **SE** | **P** | **bJ** | **bJ_se** | **pJ** |
| Signal-A | rs2205960 | 1:173191475 | G | T | 0.237 | 0.23 | 0.02 | 5.56 x 10^-28^ | 0.27 | 0.02 | 9.47 x 10^-35^ |
| Signal-B | rs6680010 | 1:173325726 | G | A | 0.336 | 0.19 | 0.02 | 6.82 x 10^-23^ | 0.22 | 0.02 | 2.21 x 10^-30^ |
| Signal-C | rs2227203 | 1:172879023 | T | C | 0.440 | -0.11 | 0.02 | 1.96 x 10^-09^ | -0.10 | 0.02 | 1.31 x 10^-07^ |
| ***TNFSF4* associations with severe COVID-19** | | | | | | | | | | | |
| **Signal number** | **SNP** | **position** | **A2** | **A1** | **freq(A1)** | **BETA** | **SE** | **P** | **bJ** | **bJ_se** | **pJ** |
| Signal-B | rs6680010 | 1:173325726 | G | A | 0.657 | -0.05 | 0.02 | 8.48 x 10^-03^ | -0.05 | 0.02 | 8.48 x 10^-03^ |
| **Table D. *TNFSF4* association results for a) SLE and b) severe COVID-19 data.** Independently associated SNPs in SLE and severe COVID-19 are displayed. * bJ, bJ_se, pJ: effect size, standard error and p-value from a joint analysis (multiple regression) of all the selected SNPs (results conditional on all other SNPs if selected from stepwise regression). | | | | | | | | | | | |

| Table E. Top 3 pathways/diseases enriched with potentially shared genes associated in the cross-trait meta-analysis (FDR < 0.01). | | | | |
| --- | --- | --- | --- | --- |
| Category | **Term ID** | **Term description** | **FDR** | **Matching proteins** |
| GO Process | GO:0006952 | Defense response | 4.62 x 10^-06^ | IL12B, IRF8, TNFSF4, CLEC1A, MAPT, ADAM15, STAT1, NCF2, STAT4, CXCR6, TYK2 |
| GO Process | GO:0019221 | Cytokine-mediated signaling pathway | 1.40 x 10^-04^ | IL12B, IL12RB2, IRF8, TNFSF4, STAT1, STAT4, CXCR6, TYK2 |
| GO Process | GO:0007166 | Cell surface receptor signaling pathway | 3.10 x 10^-04^ | IL12B, IL12RB2, IRF8, TNFSF4, CLEC1A, ADAM15,  STAT1, NCF2, STAT4, CXCR6, TYK2 |
| STRING clusters | CL:18001 | Overview of interferons-mediated signaling pathway, and Interleukin-20 family signaling | 3.70 x 10^-06^ | IL12B, IL12RB2, STAT1, STAT4, TYK2 |
| STRING clusters | CL:18088 | Positive regulation of t-helper 17 cell lineage commitment, and stat4, sh2 domain | 4.97 x 10^-05^ | IL12B, IL12RB2, STAT4 |
| Wiki­Pathways | WP5098 | T-cell activation SARS-CoV-2 | 4.90 x 10^-04^ | IL12B, IL12RB2, STAT4, TYK2 |
| Wiki­Pathways | WP2203 | Thymic stromal lymphopoietin (TSLP) signaling pathway | 2.90 x 10^-03^ | TNFSF4, STAT1, STAT4 |
| Wiki­Pathways | WP585 | Interferon type I signaling pathways | 2.90 x 10^-03^ | STAT1, STAT4, TYK2 |
| KEGG | hsa04658 | Th1 and Th2 cell differentiation | 2.89 x 10^-06^ | IL12B, IL12RB2, STAT1, STAT4, TYK2 |
| KEGG | hsa04630 | JAK-STAT signaling pathway | 2.72 x 10^-05^ | IL12B, IL12RB2, STAT1, STAT4, TYK2 |
| KEGG | hsa05321 | Inflammatory bowel disease | 2.72 x 10^-05^ | IL12B, IL12RB2, STAT1, STAT4 |
| Reactome | HSA-447115 | Interleukin-12 family signaling | 1.17 x 10^-06^ | IL12B, IL12RB2, STAT1, STAT4, TYK2 |
| Reactome | HSA-8984722 | Interleukin-35 Signalling | 1.17 x 10^-06^ | IL12RB2, STAT1, STAT4, TYK2 |
| Reactome | HSA-9020591 | Interleukin-12 signaling | 4.84 x 10^-05^ | IL12B, IL12RB2, STAT4, TYK2 |
| DISEASES | DOID:0060032 | Autoimmune disease of musculoskeletal system | 1.47 x 10^-07^ | IL12B, IRF8, TNFSF4, STAT1, NCF2, STAT4, TYK2 |
| DISEASES | DOID:417 | Autoimmune disease | 1.47 x 10^-07^ | IL12B, IL12RB2, IRF8, TNFSF4, STAT1, NCF2, STAT4, TYK2 |
| DISEASES | DOID:8857 | Lupus erythematosus | 2.10 x 10^-05^ | TNFSF4, STAT1, NCF2, STAT4 |
| See Table 1 for full list of genes. | | | | |

| ***TYK2* associations with SLE** | | | | | | | | | | | |
| --- | --- | --- | --- | --- | --- | --- | --- | --- | --- | --- | --- |
| **Signal number** | **SNP** | **position** | **A2** | **A1** | **freq(A1)** | **BETA** | **SE** | **P** | **bJ** | **bJ_se** | **pJ** |
| Signal-A | rs34536443 | 19:10463118 | G | C | 0.034 | -0.64 | 0.06 | 9.80 x 10^-26^ | -0.57 | 0.06 | 9.47 x 10^-20^ |
| Signal-B | rs34725611 | 19:10477067 | A | G | 0.275 | -0.22 | 0.02 | 3.84 x 10^-27^ | -0.16 | 0.02 | 2.86 x 10^-13^ |
| Signal-C | rs74908652 | 19:10422972 | T | C | 0.159 | -0.17 | 0.03 | 8.59 x 10^-12^ | -0.15 | 0.03 | 2.23 x 10^-08^ |
| **Results for COVID-19 lead SNPs** | | | | | | | | | | | |
| Signal-A | rs74956615 | 19:10427721 | T | A | 0.041 | -0.54 | 0.05 | 2.75 x 10^-24^ | -0.48 | 0.06 | 6.73 x 10^-18^ |
| Signal-B | rs11085727 | 19:10466123 | C | T | 0.278 | -0.22 | 0.02 | 6.92 x 10^-27^ | -0.15 | 0.02 | 2.09 x 10^-12^ |
| ***TYK2* associations with severe COVID-19** | | | | | | | | | | | |
| **Signal number** | **SNP** | **position** | **A2** | **A1** | **freq(A1)** | **BETA** | **SE** | **P** | **bJ** | **bJ_se** | **pJ** |
| Signal-A | rs74956615 | 19:10427721 | T | A | 0.047 | 0.34 | 0.05 | 3.04 x 10^-14^ | 0.26 | 0.05 | 4.06 x 10^-08^ |
| Signal-B | rs11085727 | 19:10466123 | C | T | 0.280 | 0.17 | 0.02 | 2.33 x 10^-18^ | 0.14 | 0.02 | 2.83 x 10^-12^ |
| **Results for SLE lead SNPs** | | | | | | | | | | | |
| Signal-A | rs34536443 | 19:10463118 | G | C | 0.040 | 0.39 | 0.06 | 4.89 x 10^-11^ | 0.33 | 0.06 | 7.25 x 10^-08^ |
| Signal-B | rs34725611 | 19:10477067 | A | G | 0.275 | 0.16 | 0.02 | 2.57 x 10^-16^ | 0.14 | 0.02 | 3.45 x 10^-11^ |
| Signal-C | rs74908652 | 19:10422972 | T | C | 0.166 | 0.09 | 0.03 | 5.35 x 10^-03^ | 0.07 | 0.03 | 4.13 x 10^-02^ |
| **Table F. Association results in *TYK2* locus including those for severe COVID-19 lead SNPs in SLE data and SLE lead SNPs in severe COVID-19 data.** Independently associated SNPs are displayed if more than one signal were observed. ^ϯ^ bJ, bJ_se, pJ: effect size, standard error and p-value from a joint analysis (multiple regression) of all the selected SNPs (results conditional on all other SNPs if selected from stepwise regression). | | | | | | | | | | | |

| Association and colocalisation results in *TYK2* - *PDE4A* locus | | | | | | | | | | | | |
| --- | --- | --- | --- | --- | --- | --- | --- | --- | --- | --- | --- | --- |
|  | **SNP** | **Gene** | **A1** | **freq(A1)** | **beta** | **p** | **N** | **colocalisation probability**  **(vs COVID-19)** | **colocalisation probability**  **(vs SLE)** | **bJ** | | **pJ** |
| COVID19hgA2ALL | rs11085727 | *TYK2* | T | 0.280 | 0.17 | 2.33 x 10^-18^ | 1,010,654 | 100.0% | 99.3% | 0.14 | | 2.83 x 10^-12^ |
| SLE EuroX4 | rs34725611 |  | G | 0.275 | -0.22 | 3.84 x 10^-27^ | 32,156 | 99.3% | 100.0% | -0.16 | | 2.86 x 10^-13^ |
|  | **rs11085727* |  | T | 0.278 | -0.22 | 6.92 x 10^-27^ | 32,156 |  |  | -0.15 | | 2.09 x 10^-12^ |
| eQTLGen Whole Blood | rs34725611 |  | G | 0.270 | 0.25 | 7.91 x 10^-170^ | 30,935 | 98.4% | 99.9% | 0.12 | | 4.09 x 10^-14^ |
|  | **rs11085727* |  | T | 0.276 | 0.24 | 3.60 x 10^-164^ | 30,814 |  |  | 0.10 | | 2.64 x 10^-11^ |
| GTEx v8 Whole Blood | rs11085725 |  | T | 0.308 | 0.11 | 2.73 x 10^-13^ | 558 | 99.9% | 99.9% | 0.11 | 7.98 x 10^-13^ | |
|  | **rs11085727* |  | T | 0.307 | 0.11 | 4.75 x 10^-13^ | 558 |  |  | 0.11 | 1.35 x 10^-12^ | |
| GTEx v8 Adrenal Gland | rs2304256 |  | A | 0.289 | 0.42 | 2.18 x 10^-10^ | 194 | 99.9% | 99.9% | 0.35 | | 2.27 x 10^-07^ |
|  | **rs11085727* |  | T | 0.299 | 0.41 | 4.55 x 10^-10^ | 194 |  |  | 0.34 | | 3.55 x 10^-07^ |
| COVID19hgA2ALL | rs74956615 | *PDE4A* | A | 0.047 | 0.34 | 3.04 x 10^-14^ | 1,007,034 | 100.0% | 99.1% | 0.26 | | 4.06 x 10^-08^ |
| SLE EuroX4 | rs34536443 |  | C | 0.034 | -0.64 | 9.80 x 10^-26^ | 31,840 | 99.1% | 100.0% | -0.57 | | 9.47 x 10^-20^ |
|  | **rs74956615* |  | A | 0.041 | -0.54 | 2.75 x 10^-24^ | 32,009 |  |  | -0.48 | | 6.73 x 10^-18^ |
| GTEx v8 Artery Tibial | rs144309607 |  | T | 0.053 | -0.28 | 3.02 x 10^-06^ | 476 | 99.1% | 99.7% | -0.27 | | 7.02 x 10^-06^ |
|  | **rs74956615* |  | A | 0.054 | -0.24 | 4.95 x 10^-05^ | 476 |  |  | -0.24 | | 9.48 x 10^-05^ |
| Association and colocalisation results in *CLEC1A* locus | | | | | | | | | | | | |
|  | **SNP** | **Gene** | **A1** | **freq(A1)** | **beta** | **p** | **N** | **colocalisation probability**  **(vs COVID-19)** | **colocalisation probability**  **(vs SLE)** | **bJ** | | **pJ** |
| COVID19hgA2ALL | rs7960611 | *CLEC1A* | G | 0.124 | 0.16 | 3.31 x 10^-07^ | 998,283 | 100.0% | 95.4% | 0.16 | | 3.31 x 10^-07^ |
| SLE EuroX4 | rs7960611 |  | G | 0.115 | 0.10 | 2.76 x 10^-04^ | 32,156 | 95.4% | 100.0% | 0.10 | | 2.77 x 10^-04^ |
| GTEx v8 Heart Atrial Appendage | rs2306892 |  | C | 0.110 | -0.44 | 4.50 x 10^-11^ | 320 | 96.5% | 86.9% | -0.44 | | 1.22 x 10^-10^ |
|  | **rs7960611* |  | G | 0.104 | -0.40 | 3.47 x 10^-09^ | 316 |  |  | -0.40 | | 6.34 x 10^-09^ |
| GTEx v8 Adipose Subcutaneous | rs7313750 |  | G | 0.129 | -0.22 | 9.77 x 10^-08^ | 479 | 98.2% | 90.0% | -0.22 | | 1.14 x 10^-07^ |
|  | **rs7960611* |  | G | 0.115 | -0.23 | 3.38 x 10^-07^ | 479 |  |  | -0.23 | | 4.42 x 10^-07^ |
| GTEx v8 Thyroid | rs2220436 |  | A | 0.134 | -0.27 | 7.03 x 10^-11^ | 482 | 98.4% | 90.4% | -0.27 | | 1.43 x 10^-10^ |
|  | **rs7960611* |  | G | 0.117 | -0.29 | 9.30 x 10^-11^ | 482 |  |  | -0.29 | | 1.84 x 10^-10^ |
| GTEx v8 Brain Frontal Cortex | rs7960611 |  | G | 0.118 | -0.44 | 1.49 x 10^-07^ | 157 | 99.2% | 93.2% | -0.44 | | 1.93 x 10^-07^ |
| Table G. Association and colocalisation probability results in SLE and severe COVID-19 GWAS data and eQTLGen/GTEx v8 eQTL data. Independently associated SNPs are displayed if more than one signal were observed. ^ϯ^ bJ, bJ_se, pJ: effect size, standard error and p-value from a joint analysis (multiple regression) of all the selected SNPs (results conditional on all other SNPs if selected from stepwise regression). | | | | | | | | | | | | |

| **Table H. Significant trans-pQTL (p-value < 0.01) from a pQTL analysis of associations between the SNPs in our study and the 21 previously defined IFN induced genes in a combined summary pQTL data [40, 41].** | | | | | | | | | | |
| --- | --- | --- | --- | --- | --- | --- | --- | --- | --- | --- |
| **SNP** | **chr** | **position** | **A2** | **A1** | **pQTL ID** | **pQTL gene** | **trait** | **BETA** | **SE** | **FDR** |
| signal-B  rs11085727 | 19 | 10466123 | C | T | prot-a-2701 | SERPING1 | Plasma protease C1 inhibitor | -0.10 | 0.03 | 2.25 x 10^-03^ |
|  |  |  |  |  | prot-a-738 | CXCL10 | C-X-C motif chemokine 10 | -0.09 | 0.03 | 5.40 x 10^-03^ |
| signal-B  rs34725611 | 19 | 10477067 | A | G | prot-a-738 | CXCL10 | C-X-C motif chemokine 10 | -0.09 | 0.03 | 3.49 x 10^-03^ |
|  |  |  |  |  | prot-a-2701 | SERPING1 | Plasma protease C1 inhibitor | -0.09 | 0.03 | 5.27 x 10^-03^ |
| A Bonferroni adjustment was made for multiple testing across all SNP/gene combinations. | | | | | | | | | | |

| **Table I. *TYK2* association results when running stepwise regression on the individual level SLE data using SNPTEST.** | | | | | | | | | | | |
| --- | --- | --- | --- | --- | --- | --- | --- | --- | --- | --- | --- |
| **SNP** | **position** | **A2** | **A1** | **freq(A1)** | **BETA** | **SE** | **P** | **bJ** | **bJ_se** | **pJ** | **Gene : Consequence** |
| rs34536443 | 19:10463118 | G | C | 0.03 | -0.65 | 0.06 | 3.11 x 10^-29^ | -0.62 | 0.06 | 1.65 x 10^-25^ | *TYK2* : Missense Variant (Pro > Ala) |
| rs12720356 | 19:10469975 | A | C | 0.08 | -0.25 | 0.03 | 4.17 x 10^-14^ | -0.23 | 0.04 | 1.93 x 10^-10^ | *TYK2* : Missense Variant (Ile > Ser) |
| rs280497 | 19:10464687 | A | G | 0.48 | -0.16 | 0.02 | 2.66 x 10^-19^ | -0.11 | 0.02 | 4.72 x 10^-08^ | *TYK2* : Non Coding Transcript Variant |
| rs12720358 | 19:10464540 | C | T | 0.02 | -0.25 | 0.07 | 4.85 x 10^-04^ | -0.34 | 0.07 | 1.81 x 10^-06^ | *TYK2* : Non Coding Transcript Variant |
| rs35018800 **^ϯ^** | 19:10464843 | G | A | 0.005 | -0.94 | 0.19 | 2.45 x 10^-08^ | -0.94 | 0.19 | 2.99 x 10^-08^ | *TYK2* : Missense Variant (Ala > Val) |
| *bJ, bJ_se, pJ: effect size, standard error and p-value from a joint analysis (multiple regression) of all the selected SNPs (results conditional on all other SNPs if selected from stepwise regression). ^ϯ^ An additional previously identified rare coding variant. | | | | | | | | | | | |

| **SLE associations in Hispanics - summary statistics.** | | | | | | | | | |
| --- | --- | --- | --- | --- | --- | --- | --- | --- | --- |
| **SNP** | **position** | **A2** | **A1** | **BETA** | **SE** | **P** | **cases**  **MAF** | **controls**  **MAF** | **Gene : Consequence** |
| rs34536443 | 19:10463118 | G | C | -1.08 | 0.24 | 1.28 x 10^-06^ | 0.006 | 0.021 | *TYK2* : Missense Variant (Pro > Ala) |
| rs74956615 | 19:10427721 | T | A | -0.79 | 0.21 | 5.17 x 10^-05^ | 0.009 | 0.024 | *RAVER1* : 3 Prime UTR Variant  *FDX2* : 2KB Upstream Variant |
| rs11085727 | 19:10466123 | C | T | -0.10 | 0.05 | 6.55 x 10^-02^ | 0.248 | 0.271 | *TYK2* : Intron Variant |
| rs2304256 | 19:10475652 | C | A | -0.09 | 0.06 | 1.20 x 10^-01^ | 0.188 | 0.213 | *TYK2* : Missense Variant (Val > Phe) |
| rs34725611 | 19:10477067 | A | G | -0.09 | 0.06 | 1.20 x 10^-01^ | 0.188 | 0.214 | *TYK2* : Intron Variant |
| rs74908652 | 19:10422972 | T | C | -0.16 | 0.09 | 6.41 x 10^-02^ | 0.068 | 0.089 | *FDX2* : Intron Variant |
| rs12720356 | 19:10469975 | A | C | -0.21 | 0.12 | 8.47 x 10^-02^ | 0.030 | 0.044 | *TYK2* : Missense Variant (Ile > Ser) |
| rs280497 | 19:10464687 | A | G | -0.13 | 0.05 | 4.88 x 10^-03^ | 0.391 | 0.439 | *TYK2* : Non Coding Transcript Variant |
| rs12720358 | 19:10464540 | C | T | -0.43 | 0.25 | 8.27 x 10^-02^ | 0.008 | 0.012 | *TYK2* : Non Coding Transcript Variant |
| rs35018800 | 19:10464843 | G | A | -0.41 | 0.47 | 3.73 x 10^-01^ | 0.003 | 0.004 | *TYK2* : Missense Variant (Ala > Val) |
| **SLE associations in African Americans - summary statistics.** | | | | | | | | | |
| **SNP** | **position** | **A2** | **A1** | **BETA** | **SE** | **P** | **cases**  **MAF** | **controls**  **MAF** | **Gene : Consequence** |
| rs34536443 | 19:10463118 | G | C | -0.35 | 0.31 | 2.65 x 10^-01^ | 0.005 | 0.006 | *TYK2* : Missense Variant (Pro > Ala) |
| rs74956615 | 19:10427721 | T | A | -0.17 | 0.32 | 6.06 x 10^-01^ | 0.004 | 0.005 | *RAVER1* : 3 Prime UTR Variant  *FDX2* : 2KB Upstream Variant |
| rs11085727 | 19:10466123 | C | T | -0.04 | 0.06 | 5.04 x 10^-01^ | 0.107 | 0.111 | *TYK2* : Intron Variant |
| rs2304256 | 19:10475652 | C | A | -0.03 | 0.07 | 6.57 x 10^-01^ | 0.100 | 0.103 | *TYK2* : Missense Variant (Val > Phe) |
| rs34725611 | 19:10477067 | A | G | -0.03 | 0.06 | 6.47 x 10^-01^ | 0.101 | 0.103 | *TYK2* : Intron Variant |
| rs74908652 | 19:10422972 | T | C | -0.13 | 0.07 | 6.15 x 10^-02^ | 0.077 | 0.087 | *FDX2* : Intron Variant |
| rs12720356 | 19:10469975 | A | C | -0.41 | 0.18 | 2.01 x 10^-02^ | 0.010 | 0.015 | *TYK2* : Missense Variant (Ile > Ser) |
| rs280497 | 19:10464687 | A | G | -0.04 | 0.04 | 3.52 x 10^-01^ | 0.357 | 0.349 | *TYK2* : Non Coding Transcript Variant |
| rs12720358 | 19:10464540 | C | T | -0.10 | 0.35 | 7.88 x 10^-01^ | 0.003 | 0.003 | *TYK2* : Non Coding Transcript Variant |
| rs35018800 | 19:10464843 | G | A | -0.29 | 0.49 | 5.63 x 10^-01^ | 0.002 | 0.002 | *TYK2* : Missense Variant (Ala > Val) |
| **Table J. Association results for all SNPs mentioned in paper for the Hispanic and African American Immunochip data^11^.** | | | | | | | | | |


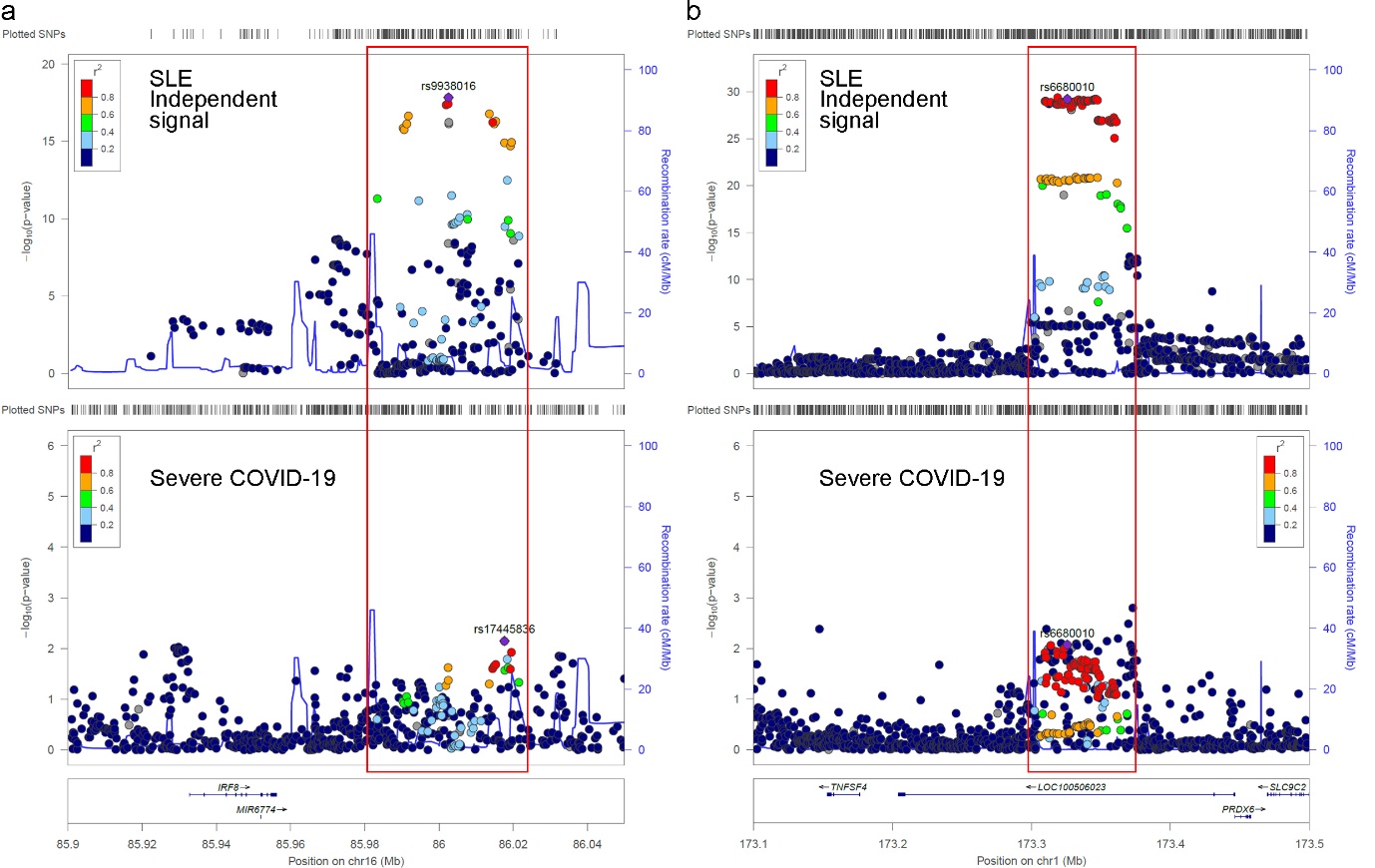


**Fig A. Locus zoom plots across a) *IRF8 and b) TNFSF4* loci for marginal associations with SLE and severe COVID-19. a) *IRF8* locus*.*** The posterior possibility (PP_H4_) for colocalisation of SLE’s signal-A with severe COVID-19 is 0.36. ^ϯ^ rs17445836 is in high LD with rs9938016 (r^2^ = 0.71). ***b) TNFSF4* locus*.*** PP_H4_ for colocalisation of SLE’s signal-B with severe COVID-19 is 0.37.The LD (r^2^ in 1000 Genome project Phase 3 EUR) is identified by color.


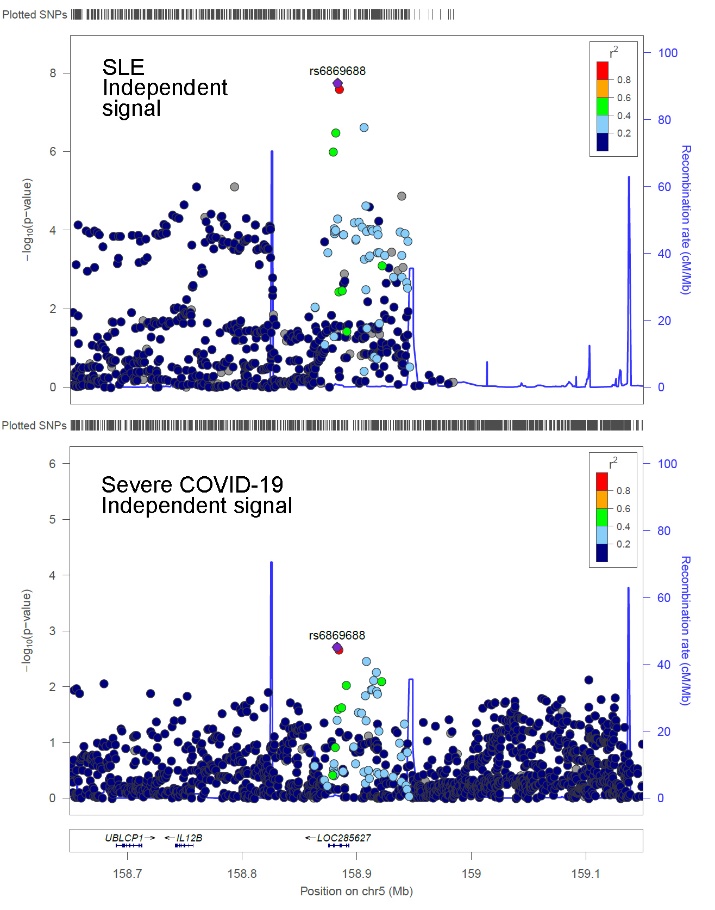


**Fig B. Chr5 *IL12B* (81.5% colocalized between independent signals)**


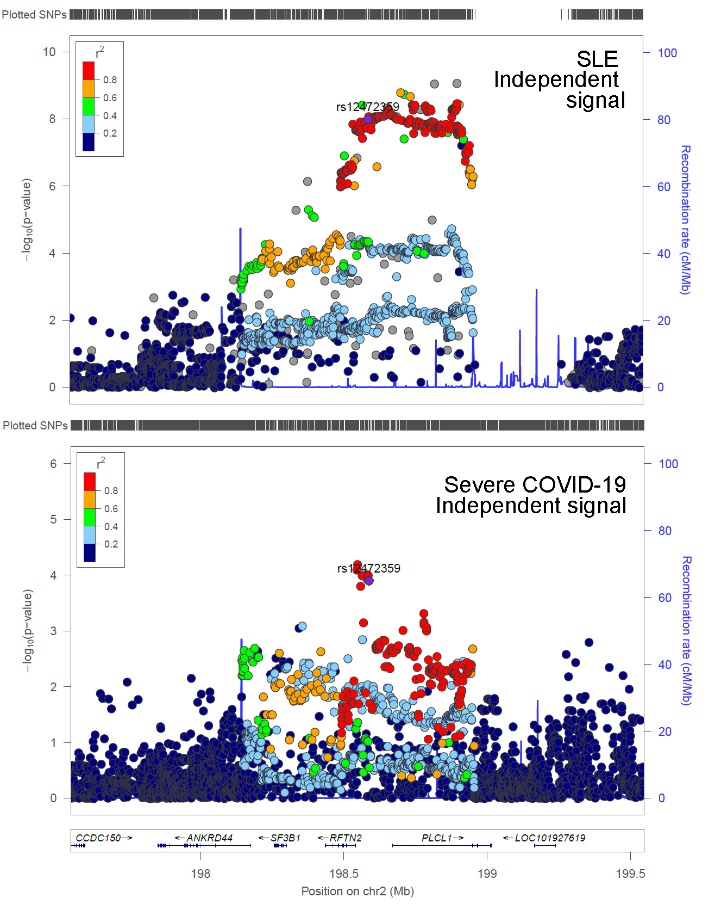


**Fig C. Chr2 *PLCL1* - *RFTN2* (80.7% colocalized between independent signals)**


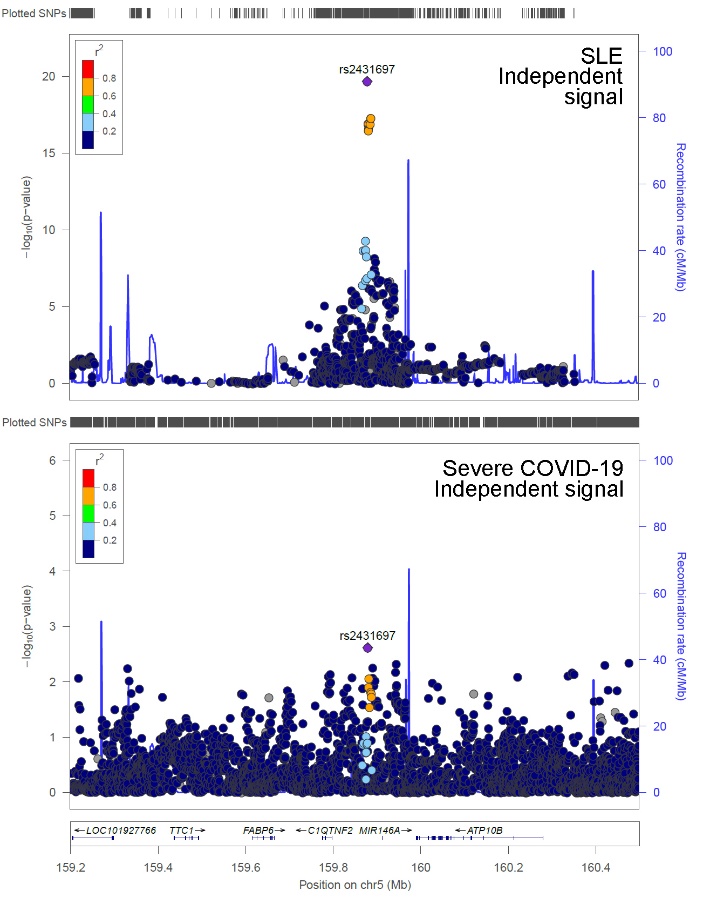


**Fig D. Chr5 *MIR146A* (80.5% colocalized between independent signals)**


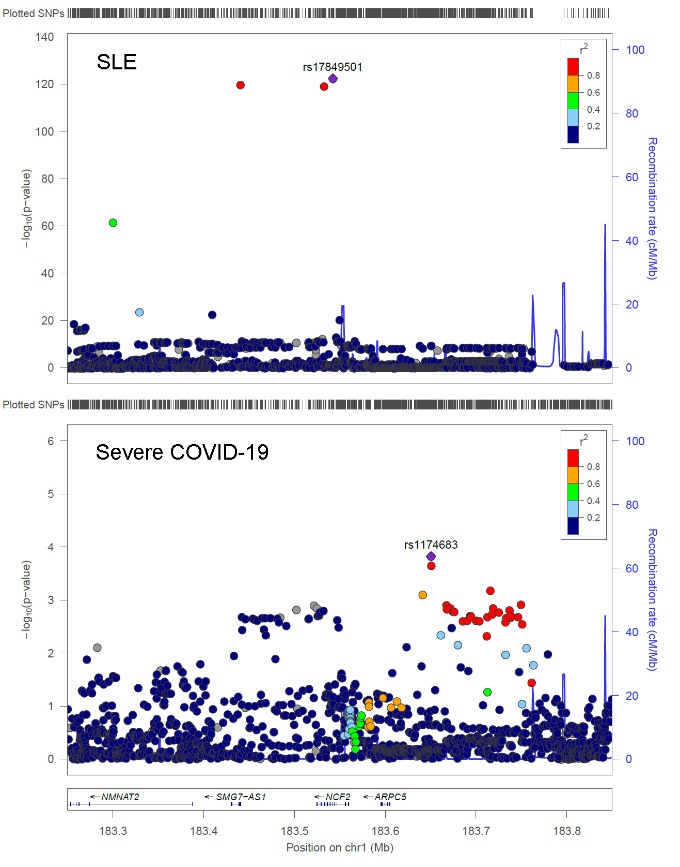

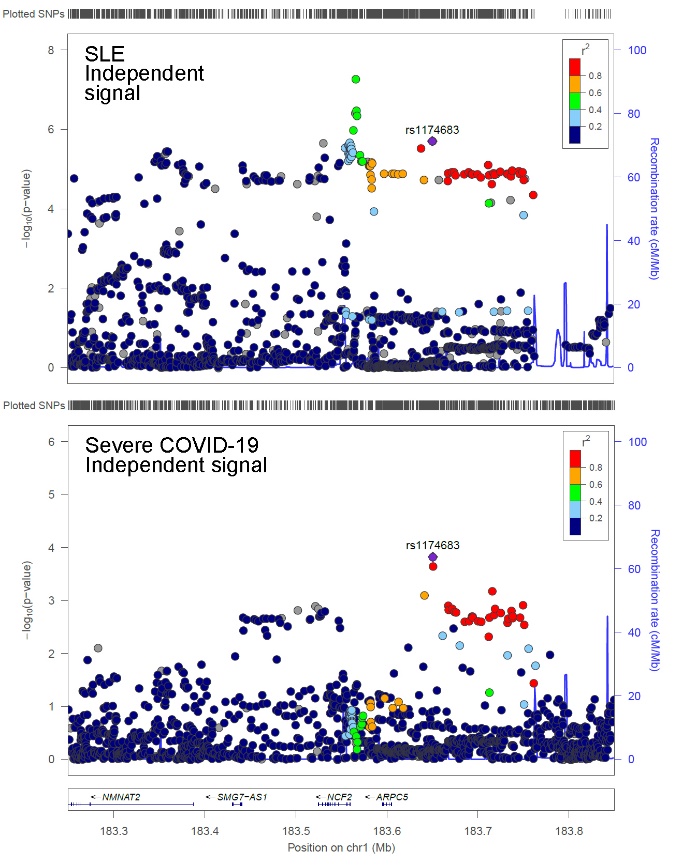


**Fig E. Chr1 *NCF2* (60.1% colocalized between independent signals)**


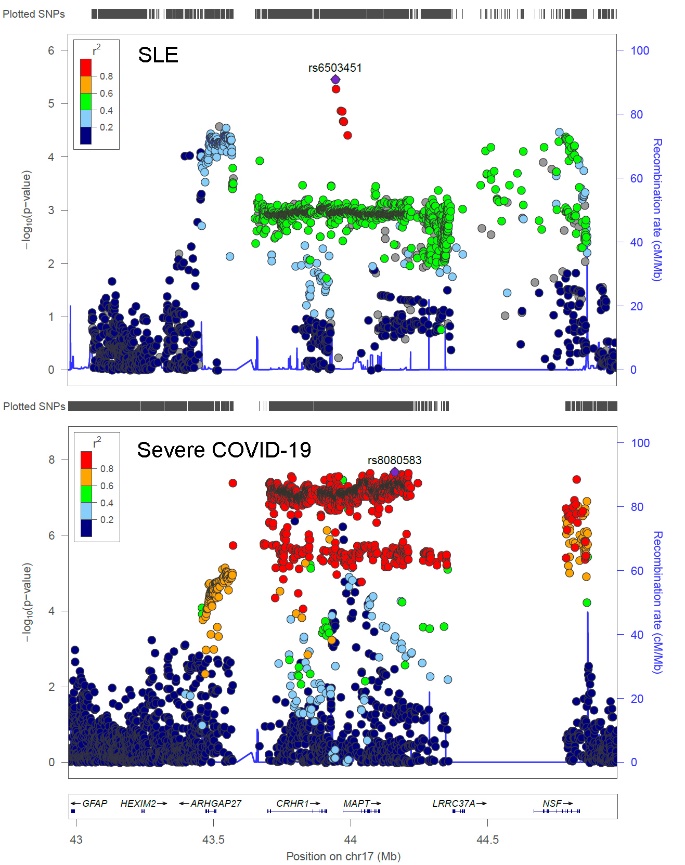

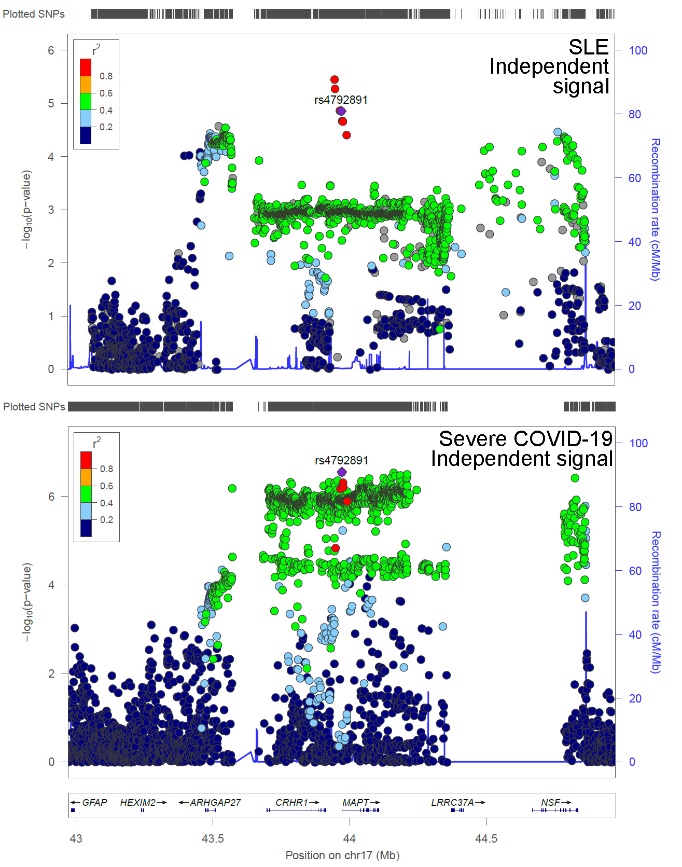


**Fig F. Chr17 *MAPT* (56.3% colocalized between independent signals)**


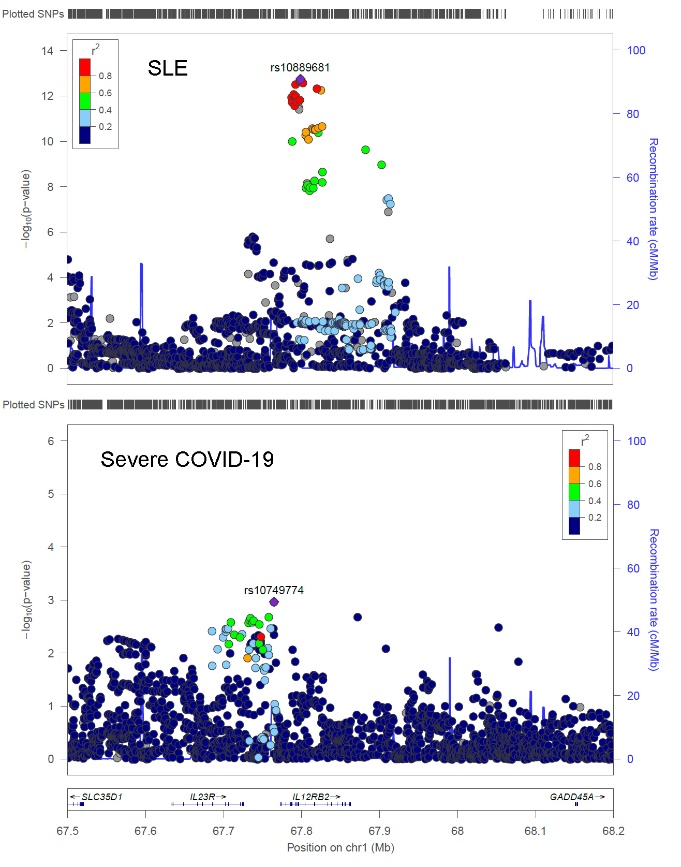

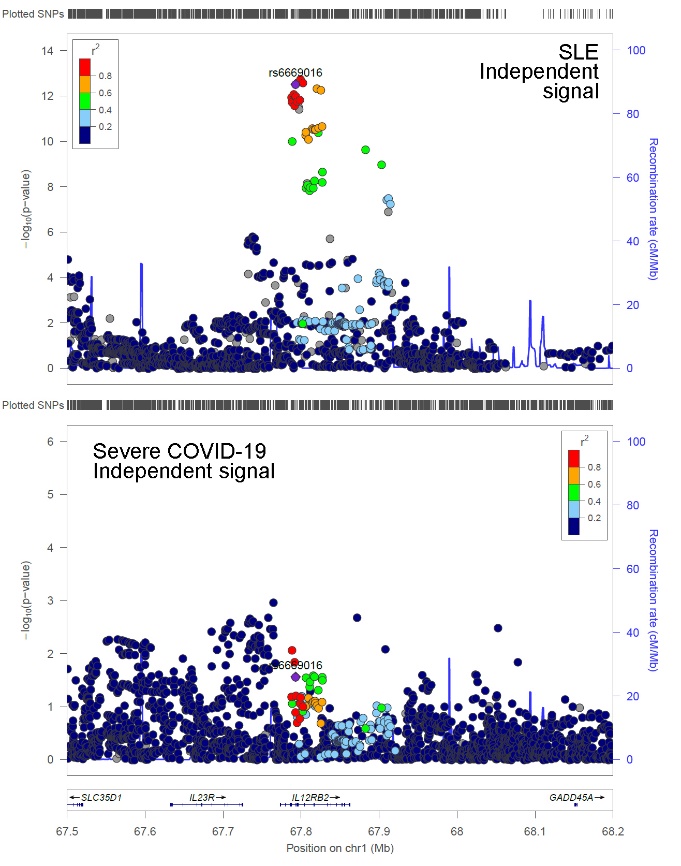


**Fig G. Chr1 *IL12RB2* (23.0% colocalized between independent signals)**


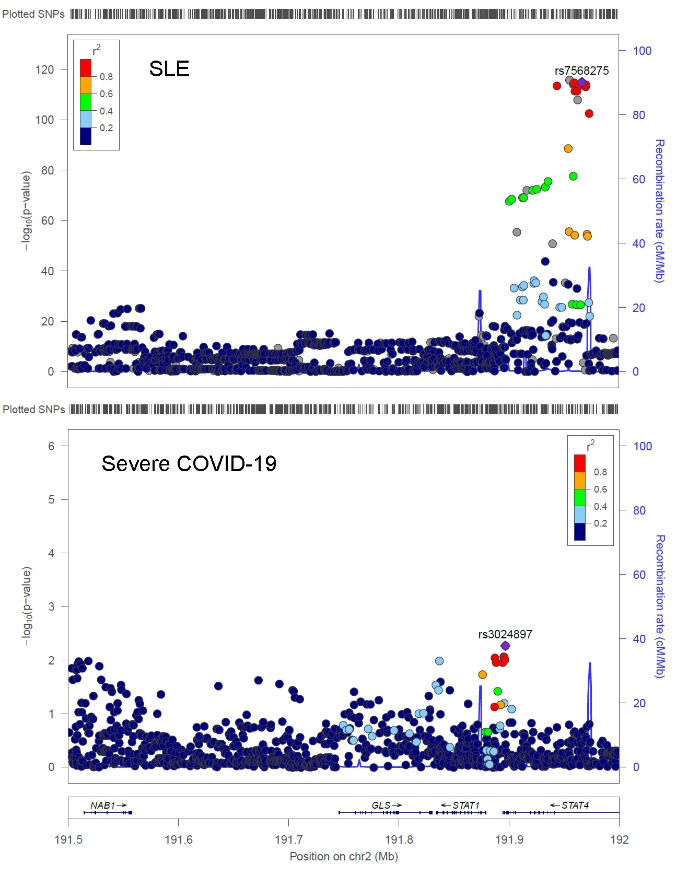

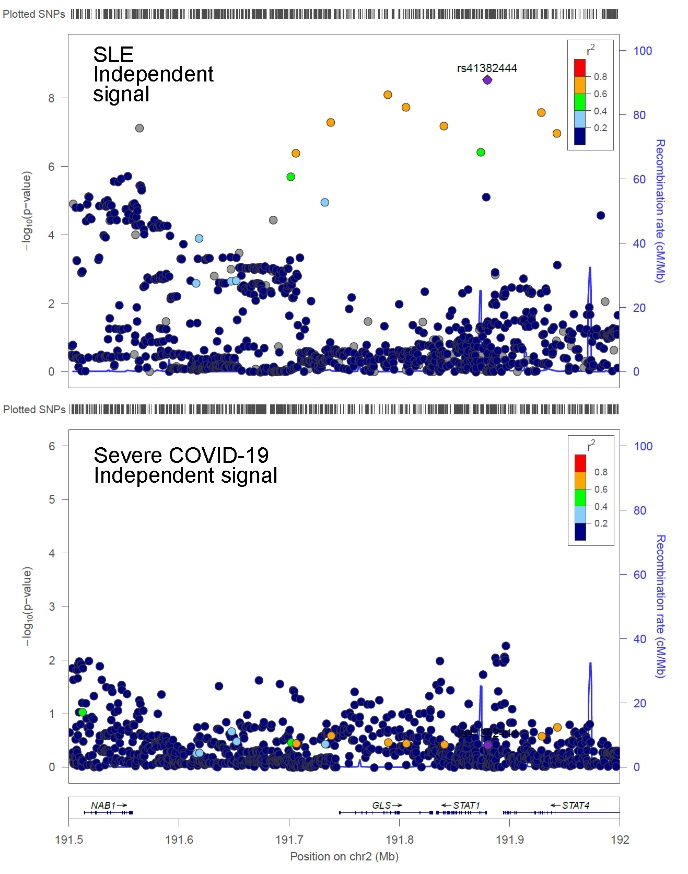


**Fig H. Chr2 *STAT1* - *STAT4* (14.6% colocalized between independent signals)**


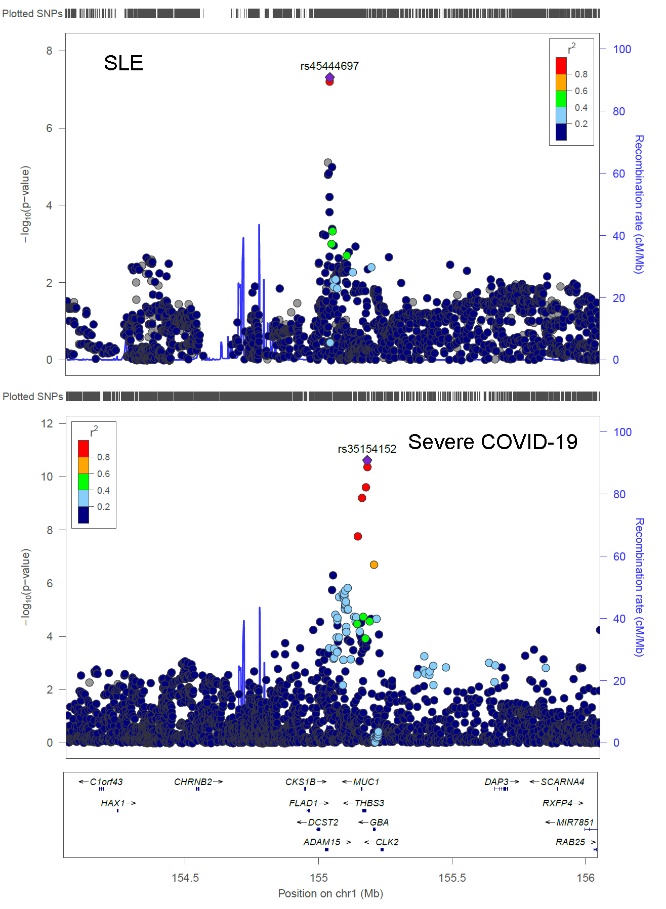

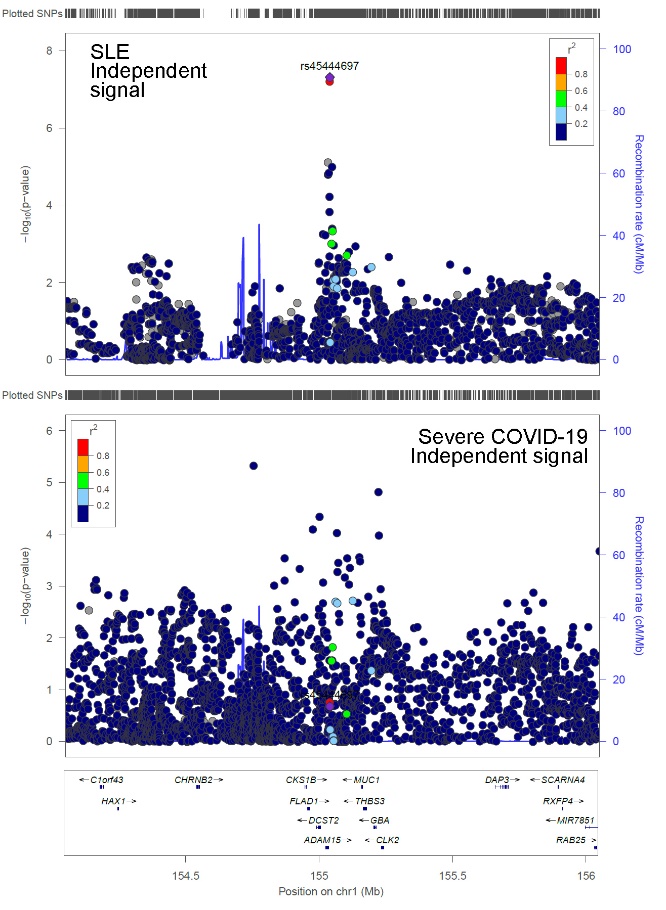


**Fig I. Chr1 *ADAM15* (8.01% colocalized between independent signals)**


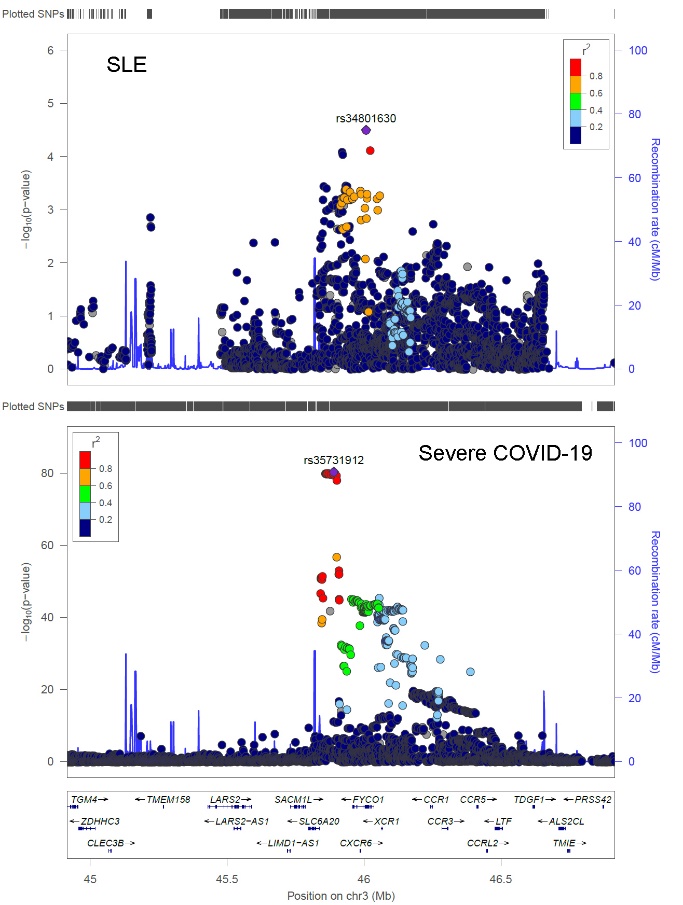

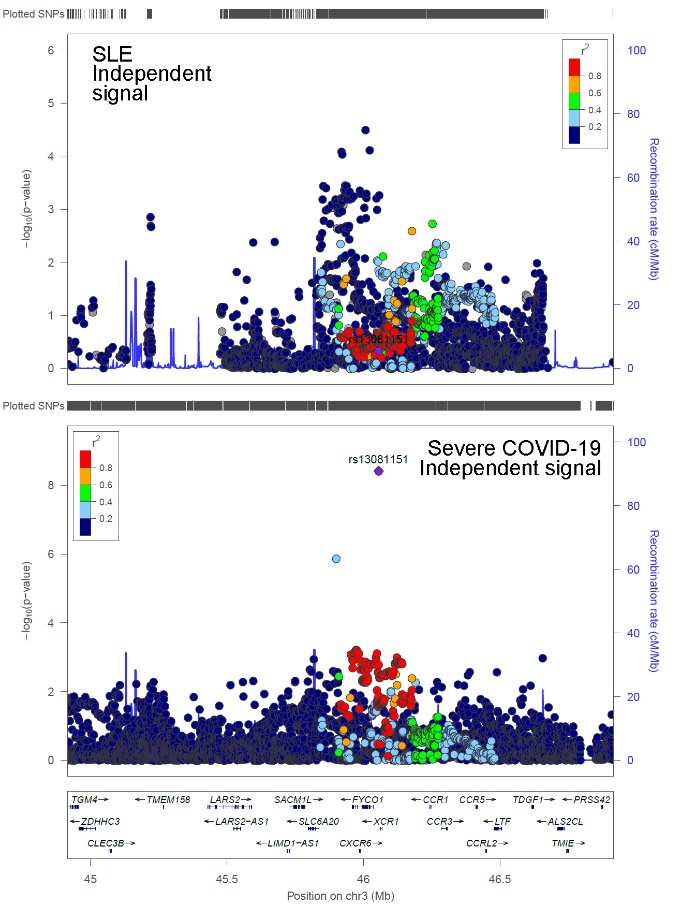


**Fig J. Chr3 *CXCR6* (6.03% colocalized between independent signals)**


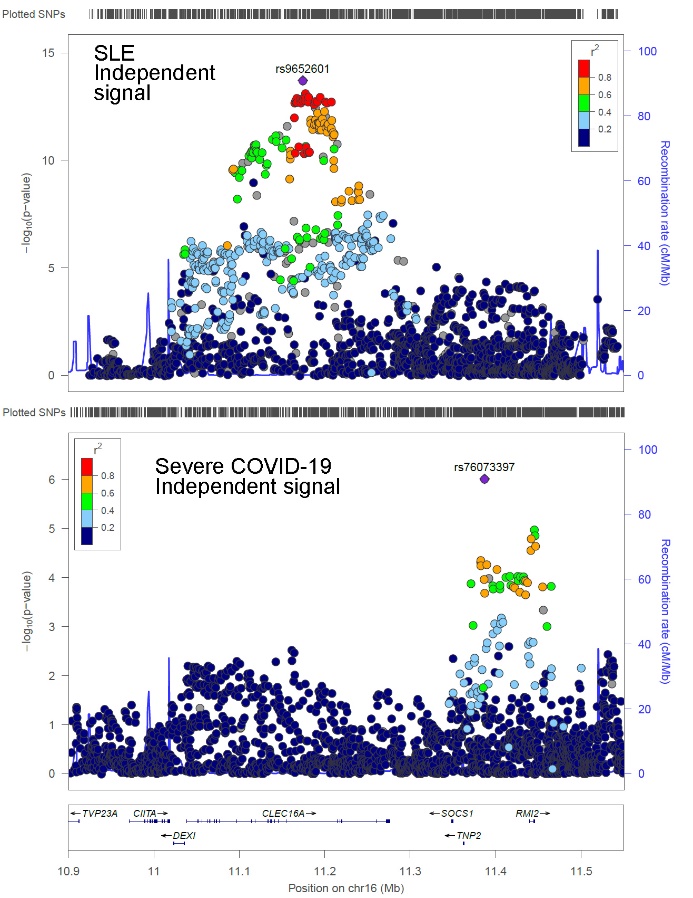


**Fig K. Chr16 *RMI2* (1.18% colocalized between independent signals)**


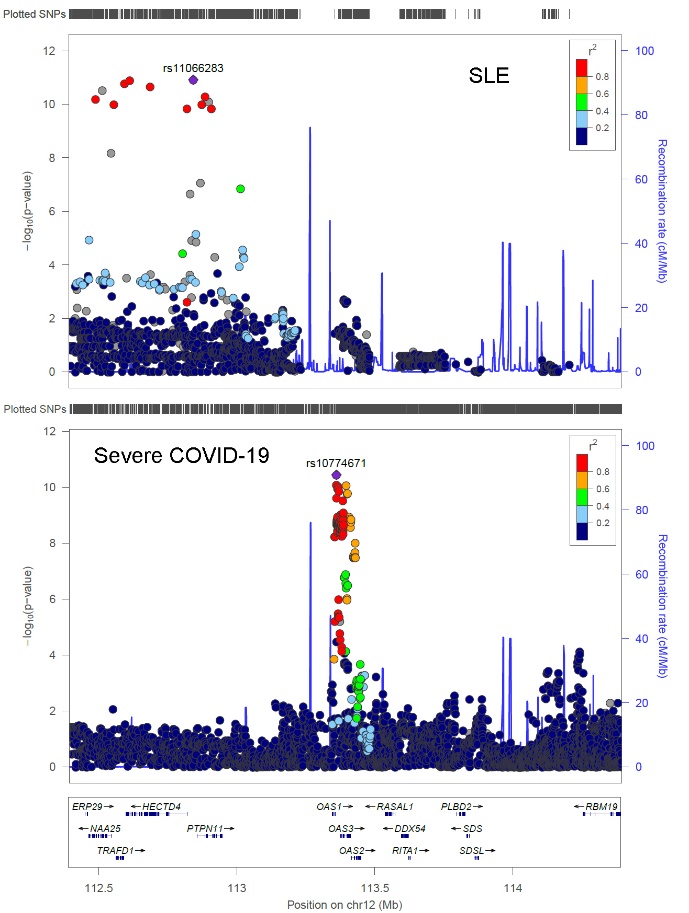

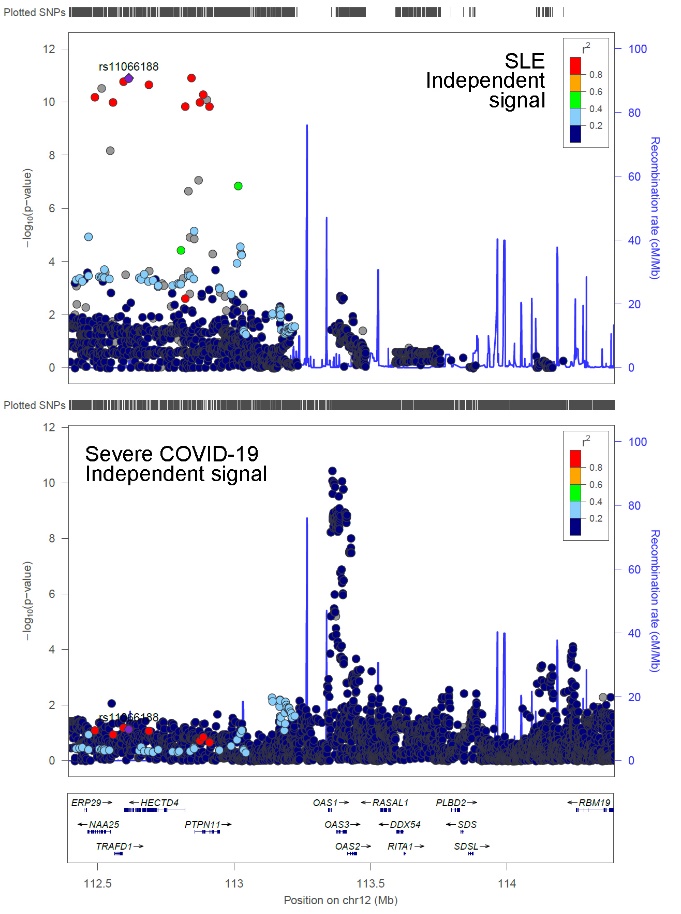


**Fig L. Chr12 *OAS1* - *OAS3* (0.00% colocalized between independent signals)**


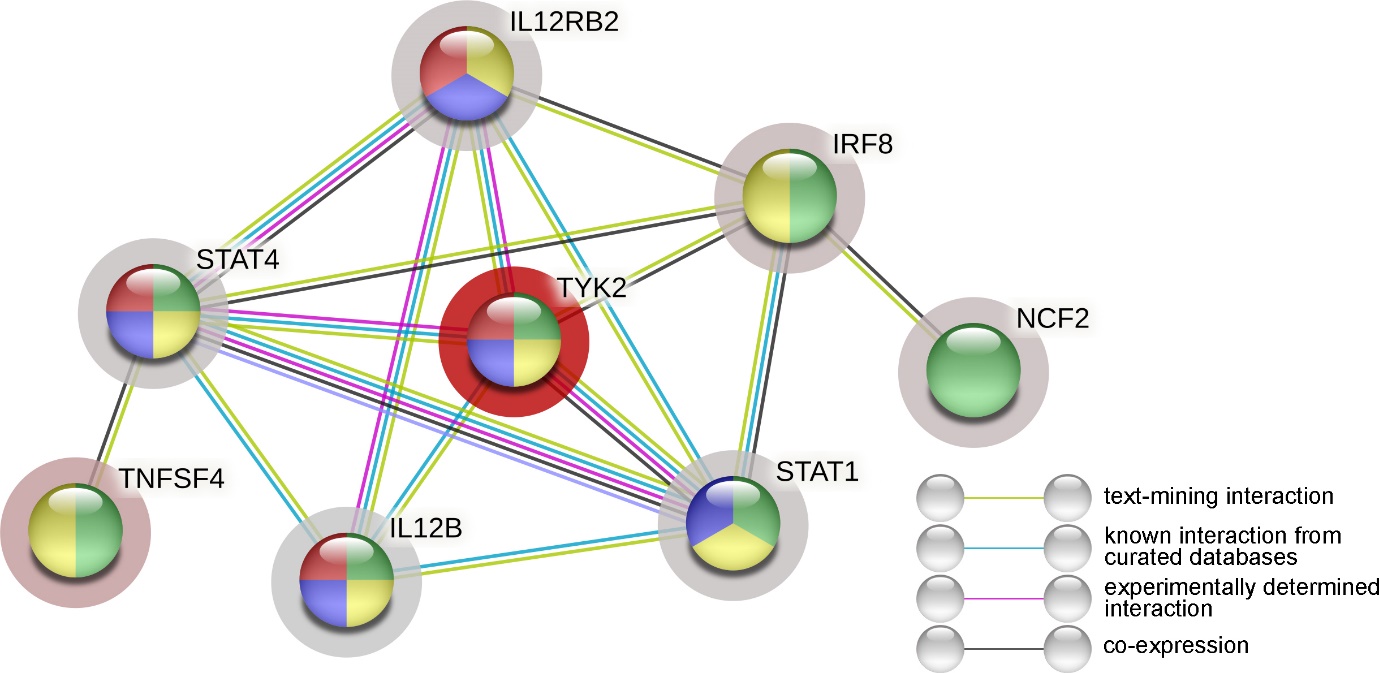


**Fig M. A gene sub-network from P-value ranked cross-trait meta-analysis association results of severe COVID-19 and SLE.** P-value < 5 x 10^-08^, disconnected nodes in the network were hidden. The halo color of nodes is based the log_10_(P-value of cross-trait meta-analysis). The nodes in blue indicate genes involved in Overview of interferons-mediated signaling pathway, and Interleukin-20 family signaling (CL:18001, FDR = 3.70 x 10^-06^), the nodes in red indicate genes involved in T-cell activation SARS-CoV-2 (WP5098, FDR = 4.90 x 10^-04^), the nodes in green indicate genes involved in Defense response (GO:0006952, FDR = 4.62 x 10^-06^), and the nodes in yellow indicate genes involved in Cytokine-mediated signaling pathway (GO:0019221, FDR = 1.40 x 10^-04^).


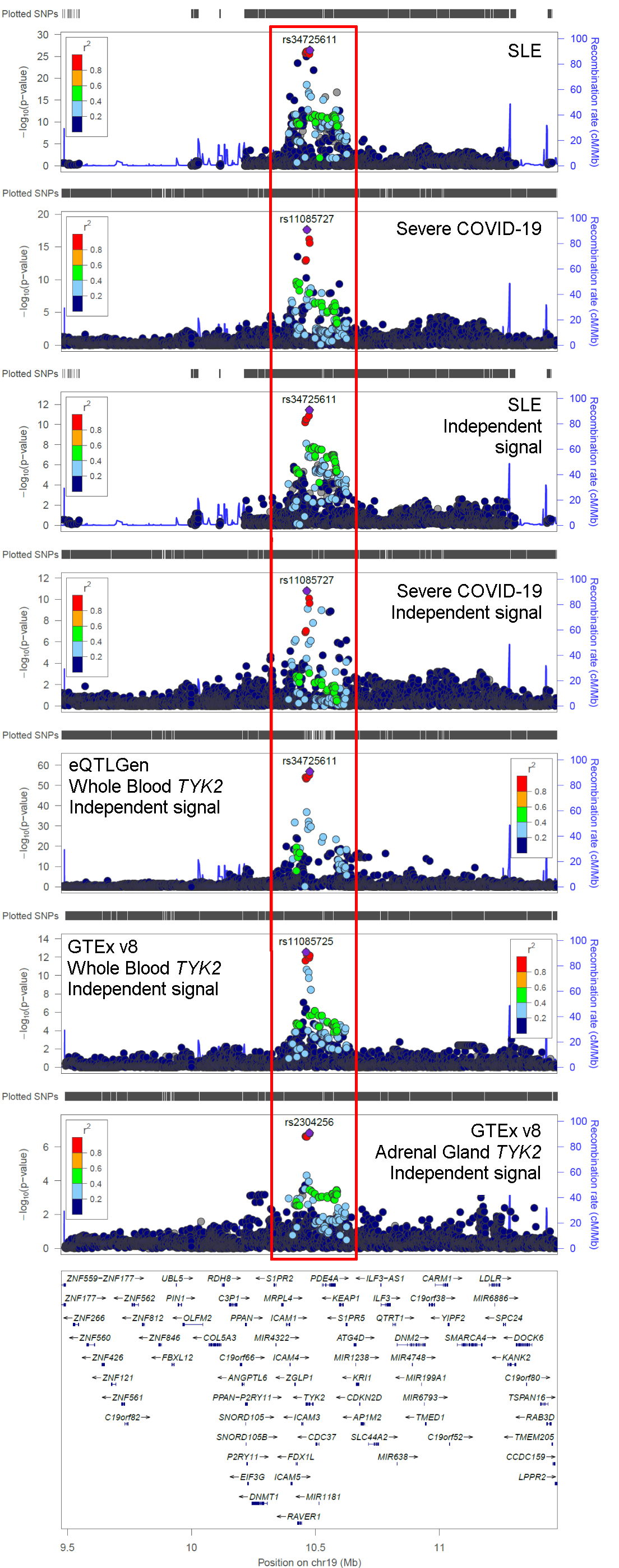


**Fig N. Full set of eQTL colocalisation with *TYK2* genetic association signals.**


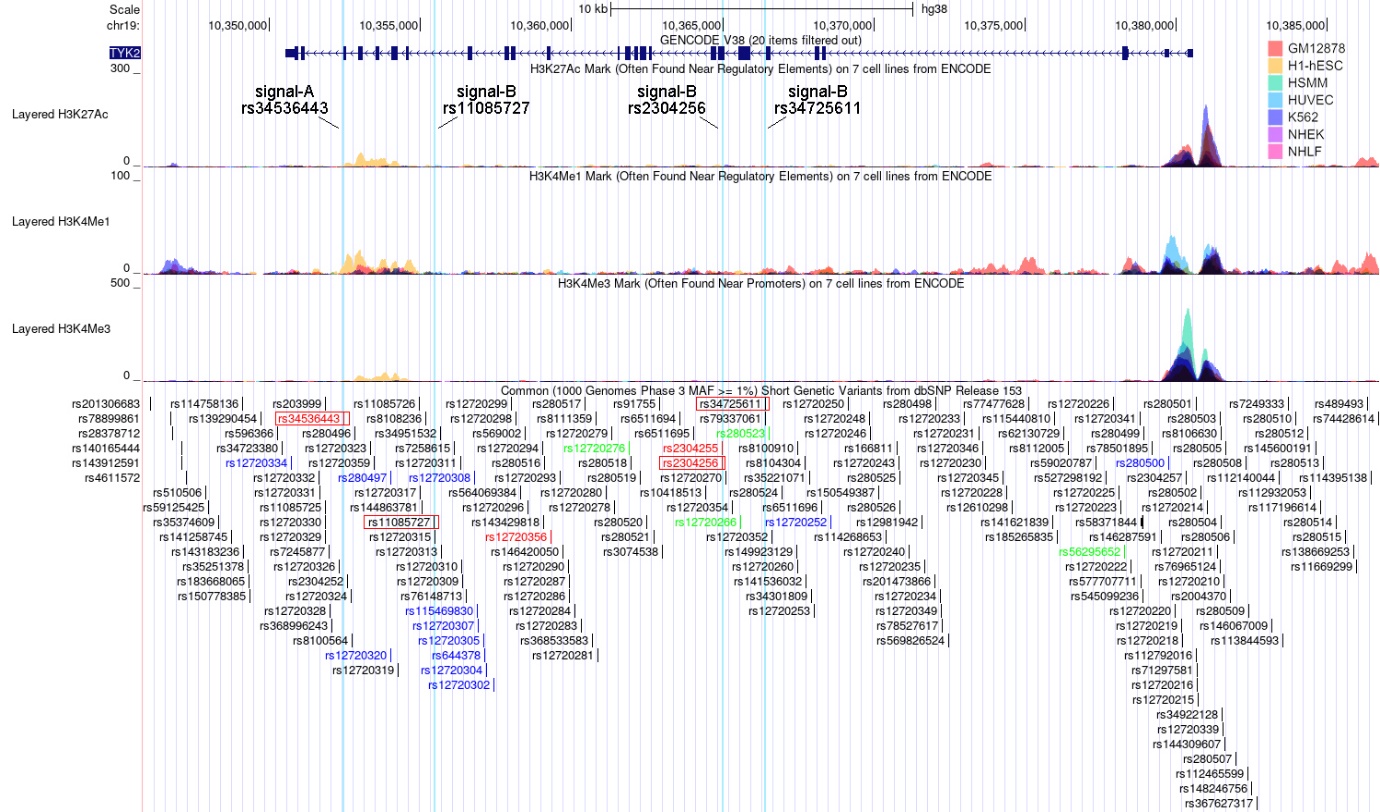


**Fig O. Epigenetic modification at *TYK2* locus.** *TYK2* associated variants are highlighted with blue lines and red boxes. Protein-altering variants and splice site variants are red. Synonymous codon variants are green. Non-coding transcript or Untranslated Region (UTR) variants are blue. Different cell lines are identified by indicated colors (H1-hESC: H1 human embryonic stem cell line, GM12878: LCL cell line from B-lymphocyte).


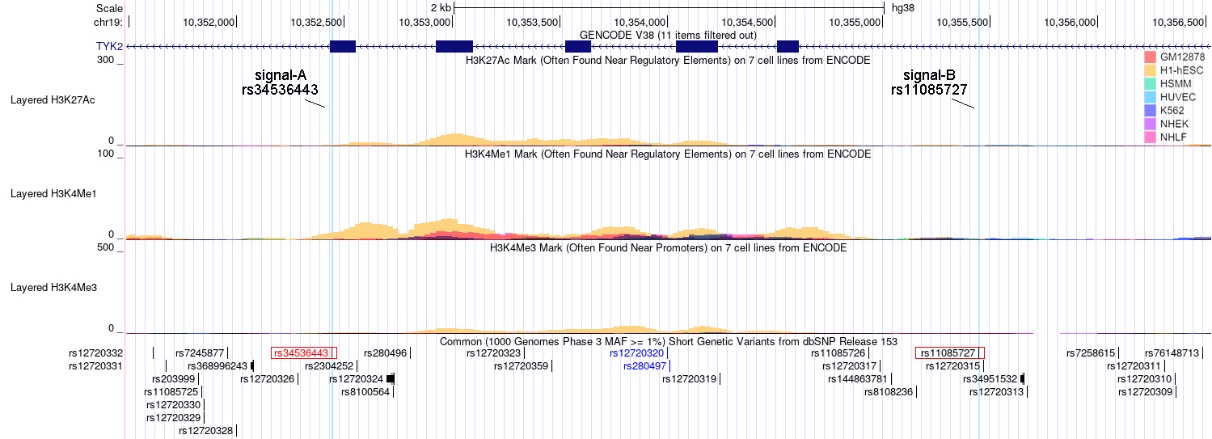


**Fig P. Epigenetic modification at *TYK2* locus (chr19: 10,351,500 – 10,356,500 bp).** *TYK2* associated variants are highlighted with blue lines and red boxes. Protein-altering variants and splice site variants are red. Non-coding transcript or Untranslated Region (UTR) variants are blue. Different cell lines are identified by indicated colors (H1-hESC: H1 human embryonic stem cell line).


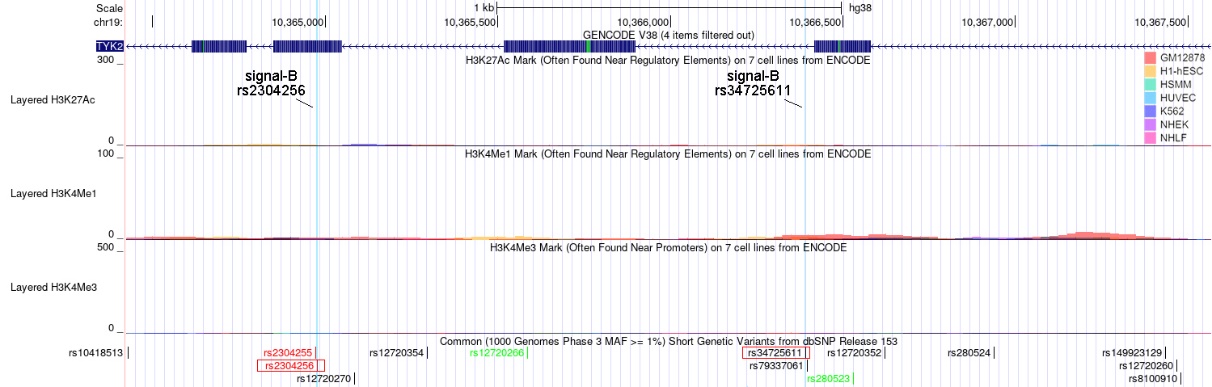


**Fig Q. Epigenetic modification at *TYK2* locus (chr19:10,364,500 – 10,367,500 bp).** *TYK2* associated variants are highlighted with blue lines and red boxes. Protein-altering variants and splice site variants are red. Synonymous codon variants are green. Different cell lines are identified by indicated colors (GM12878: LCL cell line from B-lymphocyte).


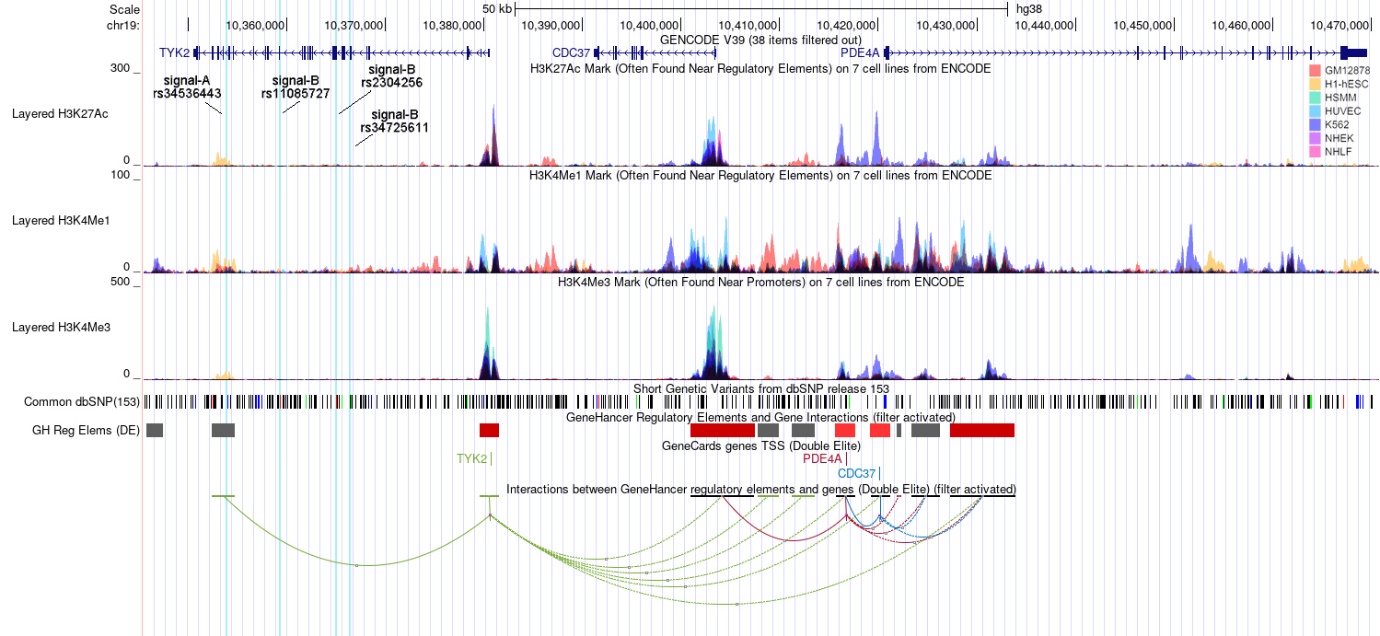


**Fig R. Epigenetic modification and chromatin looping at *TYK2* locus.** *TYK2* associated variants are highlighted with blue lines. Protein-altering variants and splice site variants are red. Synonymous codon variants are green. Non-coding transcript or Untranslated Region (UTR) variants are blue. Different cell lines are identified by indicated colors (H1-hESC: H1 human embryonic stem cell line). Regulatory elements and interactions between regulatory elements were annotated by data from GeneHancers database (embedded in GeneCards). Colors are used to distinguish promoters (red) and enhancers (grey) and darker color indicates higher GeneHancer element confidence score. The curves show interactions between regulatory elements (only interactions with both ends in window were shown). Interactions in the reverse direction (Gene transcription start sites (TSS) precedes GeneHancer element data on the plot) are drawn with a dashed line.


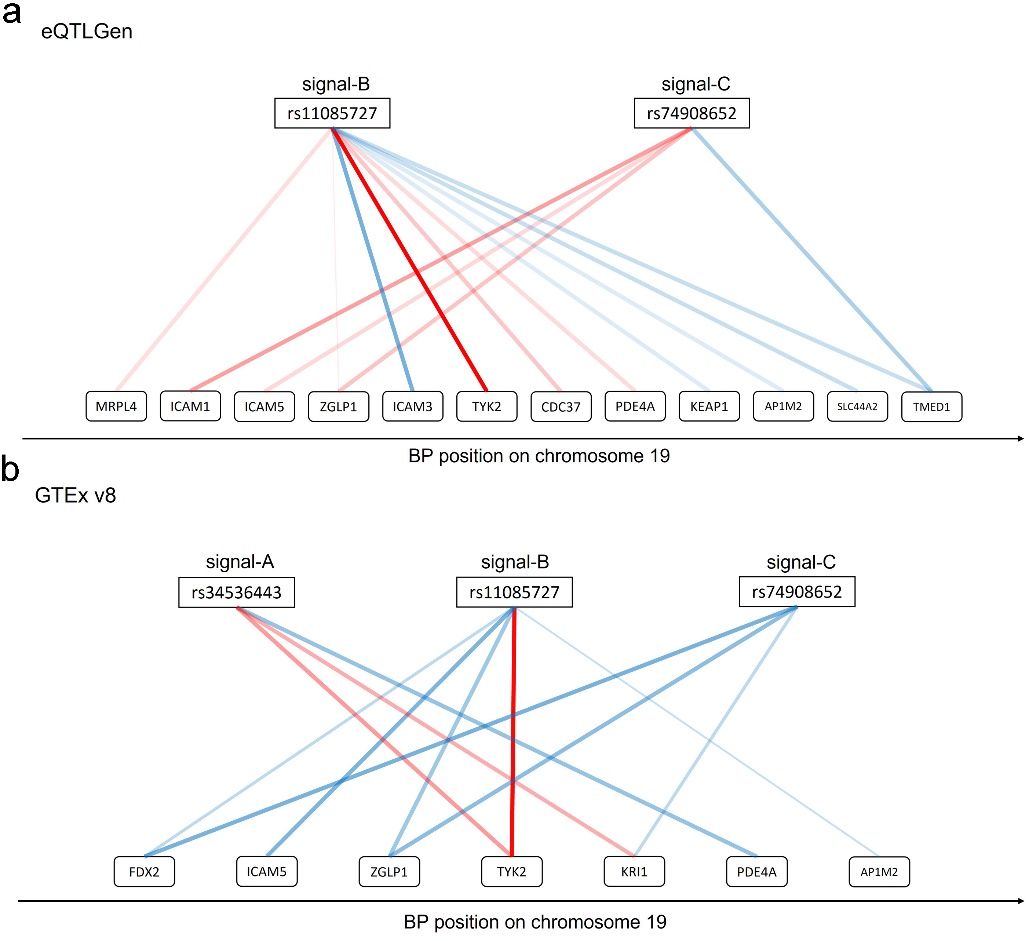


**Fig S. (a, b) Graphic demonstrating the lead eQTL SNPs at *TYK2* described in the manuscript and the genes they are eQTL for.** Genes are listed in order of base pair position on chromosome 19. The width and transparency of lines represents the level of significance (-log10 p-value range: 4.8 – 163.4 in eQTLGen, 3.8 – 15.6 in GTEx v8). The red/blue lines show the direction of effect on gene expression: red means increase, blue means decrease.


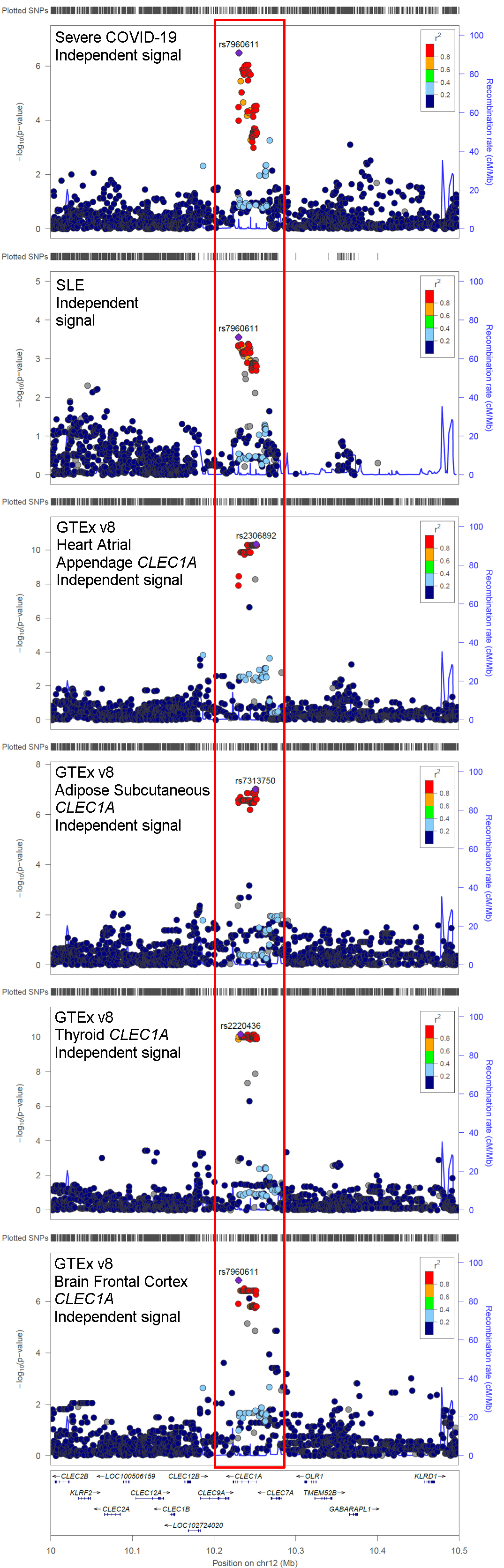


**Fig T. Full set of eQTL colocalisation with *CLEC1A* genetic association signals.**


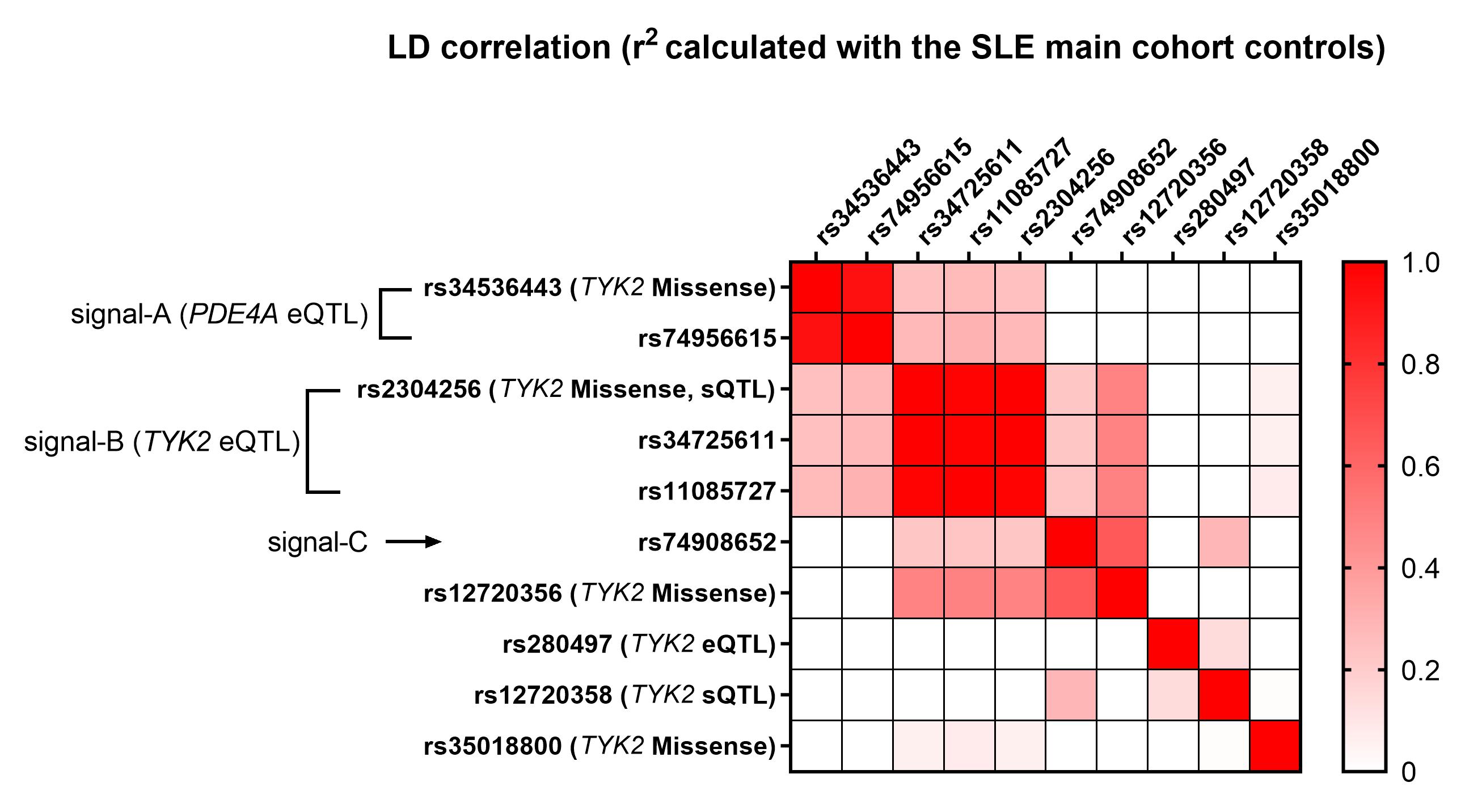


**Fig U. LD correlation matrix of lead SNPs in *TYK2* locus**

**References**

1. Dendrou CA, Cortes A, Shipman L, Evans HG, Attfield KE, Jostins L, et al. Resolving TYK2 locus genotype-to-phenotype differences in autoimmunity. Sci Transl Med. 2016;8(363):363ra149. Epub 2016/11/04. doi: 10.1126/scitranslmed.aag1974. PubMed PMID: 27807284; PubMed Central PMCID: PMCPMC5737835.

2. Diogo D, Bastarache L, Liao KP, Graham RR, Fulton RS, Greenberg JD, et al. TYK2 protein-coding variants protect against rheumatoid arthritis and autoimmunity, with no evidence of major pleiotropic effects on non-autoimmune complex traits. PLoS One. 2015;10(4):e0122271. Epub 2015/04/08. doi: 10.1371/journal.pone.0122271. PubMed PMID: 25849893; PubMed Central PMCID: PMCPMC4388675.

3. Võsa Uea. Unraveling the polygenic architecture of complex traits using blood eQTL meta-analysis. bioRxiv Preprint at, <http://biorxivorg/content/early/2018/10/19/447367abstract> (2018).

4. Lepik K, Annilo T, Kukuskina V, e QC, Kisand K, Kutalik Z, et al. C-reactive protein upregulates the whole blood expression of CD59 - an integrative analysis. PLoS Comput Biol. 2017;13(9):e1005766. Epub 2017/09/19. doi: 10.1371/journal.pcbi.1005766. PubMed PMID: 28922377; PubMed Central PMCID: PMCPMC5609773.

5. Lopez-Isac E, Campillo-Davo D, Bossini-Castillo L, Guerra SG, Assassi S, Simeon CP, et al. Influence of TYK2 in systemic sclerosis susceptibility: a new locus in the IL-12 pathway. Ann Rheum Dis. 2016;75(8):1521-6. Epub 2015/09/05. doi: 10.1136/annrheumdis-2015-208154. PubMed PMID: 26338038; PubMed Central PMCID: PMCPMC7228811.

6. Dand N, Mucha S, Tsoi LC, Mahil SK, Stuart PE, Arnold A, et al. Exome-wide association study reveals novel psoriasis susceptibility locus at TNFSF15 and rare protective alleles in genes contributing to type I IFN signalling. Hum Mol Genet. 2017;26(21):4301-13. Epub 2017/10/04. doi: 10.1093/hmg/ddx328. PubMed PMID: 28973304; PubMed Central PMCID: PMCPMC5886170.

7. Westra HJ, Martinez-Bonet M, Onengut-Gumuscu S, Lee A, Luo Y, Teslovich N, et al. Fine-mapping and functional studies highlight potential causal variants for rheumatoid arthritis and type 1 diabetes. Nat Genet. 2018;50(10):1366-74. Epub 2018/09/19. doi: 10.1038/s41588-018-0216-7. PubMed PMID: 30224649; PubMed Central PMCID: PMCPMC6364548.

8. Harley JB, Alarcon-Riquelme ME, Criswell LA, Jacob CO, Kimberly RP, Moser KL, et al. Genome-wide association scan in women with systemic lupus erythematosus identifies susceptibility variants in ITGAM, PXK, KIAA1542 and other loci. Nat Genet. 2008;40(2):204-10. doi: 10.1038/ng.81. PubMed PMID: WOS:000252732900019.

9. Hom G, Graham RR, Modrek B, Taylor KE, Ortmann W, Garnier S, et al. Association of systemic lupus erythematosus with C8orf13-BLK and ITGAM-ITGAX. N Engl J Med. 2008;358(9):900-9. doi: 10.1056/NEJMoa0707865. PubMed PMID: 18204098.

10. Bentham J, Morris DL, Cunninghame Graham DS, Pinder CL, Tombleson P, Behrens TW, et al. Genetic association analyses implicate aberrant regulation of innate and adaptive immunity genes in the pathogenesis of systemic lupus erythematosus. Nat Genet. 2015;47(12):1457-64. doi: 10.1038/ng.3434. PubMed PMID: 26502338; PubMed Central PMCID: PMCPMC4668589.

11. Langefeld CD, Ainsworth HC, Cunninghame Graham DS, Kelly JA, Comeau ME, Marion MC, et al. Transancestral mapping and genetic load in systemic lupus erythematosus. Nat Commun. 2017;8:16021. doi: 10.1038/ncomms16021. PubMed PMID: 28714469; PubMed Central PMCID: PMCPMC5520018.

12. Pairo-Castineira E, Clohisey S, Klaric L, Bretherick AD, Rawlik K, Pasko D, et al. Genetic mechanisms of critical illness in COVID-19. Nature. 2021;591(7848):92-8. Epub 2020/12/12. doi: 10.1038/s41586-020-03065-y. PubMed PMID: 33307546.

13. COVID-19 Host Genetics Initiative, Ganna A. Mapping the human genetic architecture of COVID-19: an update. medRxiv. 2022:2021.11.08.21265944. doi: 10.1101/2021.11.08.21265944.

14. Acosta-Herrera M, Kerick M, Gonzalez-Serna D, Myositis Genetics C, Scleroderma Genetics C, Wijmenga C, et al. Genome-wide meta-analysis reveals shared new loci in systemic seropositive rheumatic diseases. Ann Rheum Dis. 2019;78(3):311-9. Epub 2018/12/24. doi: 10.1136/annrheumdis-2018-214127. PubMed PMID: 30573655; PubMed Central PMCID: PMCPMC6800208.

15. Morris DL, Sheng YJ, Zhang Y, Wang YF, Zhu ZW, Tombleson P, et al. Genome-wide association meta-analysis in Chinese and European individuals identifies ten new loci associated with systemic lupus erythematosus. Nat Genet. 2016;48(8):940-+. doi: 10.1038/ng.3603. PubMed PMID: WOS:000380755100019.

16. Marquez A, Vidal-Bralo L, Rodriguez-Rodriguez L, Gonzalez-Gay MA, Balsa A, Gonzalez-Alvaro I, et al. A combined large-scale meta-analysis identifies COG6 as a novel shared risk locus for rheumatoid arthritis and systemic lupus erythematosus. Ann Rheum Dis. 2017;76(1):286-94. Epub 2016/05/20. doi: 10.1136/annrheumdis-2016-209436. PubMed PMID: 27193031.

17. Li YR, Li J, Zhao SD, Bradfield JP, Mentch FD, Maggadottir SM, et al. Meta-analysis of shared genetic architecture across ten pediatric autoimmune diseases. Nat Med. 2015;21(9):1018-27. Epub 2015/08/25. doi: 10.1038/nm.3933. PubMed PMID: 26301688; PubMed Central PMCID: PMCPMC4863040.

18. Kousathanas A, Pairo-Castineira E, Rawlik K, Stuckey A, Odhams CA, Walker S, et al. Whole genome sequencing reveals host factors underlying critical Covid-19. Nature. 2022. Epub 2022/03/08. doi: 10.1038/s41586-022-04576-6. PubMed PMID: 35255492.

19. Shelton JF, Shastri AJ, Ye C, Weldon CH, Filshtein-Sonmez T, Coker D, et al. Trans-ancestry analysis reveals genetic and nongenetic associations with COVID-19 susceptibility and severity. Nat Genet. 2021;53(6):801-8. Epub 2021/04/24. doi: 10.1038/s41588-021-00854-7. PubMed PMID: 33888907.

20. Yin X, Kim K, Suetsugu H, Bang SY, Wen L, Koido M, et al. Meta-analysis of 208370 East Asians identifies 113 susceptibility loci for systemic lupus erythematosus. Ann Rheum Dis. 2021;80(5):632-40. Epub 2020/12/05. doi: 10.1136/annrheumdis-2020-219209. PubMed PMID: 33272962; PubMed Central PMCID: PMCPMC8053352.

21. Sakaue S, Kanai M, Tanigawa Y, Karjalainen J, Kurki M, Koshiba S, et al. A cross-population atlas of genetic associations for 220 human phenotypes. Nat Genet. 2021;53(10):1415-24. Epub 2021/10/02. doi: 10.1038/s41588-021-00931-x. PubMed PMID: 34594039.

22. Tangtanatakul P, Thumarat C, Satproedprai N, Kunhapan P, Chaiyasung T, Klinchanhom S, et al. Meta-analysis of genome-wide association study identifies FBN2 as a novel locus associated with systemic lupus erythematosus in Thai population. Arthritis Res Ther. 2020;22(1):185. Epub 2020/08/11. doi: 10.1186/s13075-020-02276-y. PubMed PMID: 32771030; PubMed Central PMCID: PMCPMC7414652.

23. Demirci FY, Wang X, Kelly JA, Morris DL, Barmada MM, Feingold E, et al. Identification of a New Susceptibility Locus for Systemic Lupus Erythematosus on Chromosome 12 in Individuals of European Ancestry. Arthritis Rheumatol. 2016;68(1):174-83. Epub 2015/09/01. doi: 10.1002/art.39403. PubMed PMID: 26316170; PubMed Central PMCID: PMCPMC4747422.

24. Armstrong DL, Zidovetzki R, Alarcon-Riquelme ME, Tsao BP, Criswell LA, Kimberly RP, et al. GWAS identifies novel SLE susceptibility genes and explains the association of the HLA region. Genes Immun. 2014;15(6):347-54. Epub 2014/05/30. doi: 10.1038/gene.2014.23. PubMed PMID: 24871463; PubMed Central PMCID: PMCPMC4156543.

25. Yang W, Tang H, Zhang Y, Tang X, Zhang J, Sun L, et al. Meta-analysis followed by replication identifies loci in or near CDKN1B, TET3, CD80, DRAM1, and ARID5B as associated with systemic lupus erythematosus in Asians. Am J Hum Genet. 2013;92(1):41-51. Epub 2013/01/01. doi: 10.1016/j.ajhg.2012.11.018. PubMed PMID: 23273568; PubMed Central PMCID: PMCPMC3542470.

26. Chung SA, Taylor KE, Graham RR, Nititham J, Lee AT, Ortmann WA, et al. Differential genetic associations for systemic lupus erythematosus based on anti-dsDNA autoantibody production. PLoS Genet. 2011;7(3):e1001323. Epub 2011/03/17. doi: 10.1371/journal.pgen.1001323. PubMed PMID: 21408207; PubMed Central PMCID: PMCPMC3048371.

27. Han JW, Zheng HF, Cui Y, Sun LD, Ye DQ, Hu Z, et al. Genome-wide association study in a Chinese Han population identifies nine new susceptibility loci for systemic lupus erythematosus. Nat Genet. 2009;41(11):1234-7. Epub 2009/10/20. doi: 10.1038/ng.472. PubMed PMID: 19838193.

28. Gateva V, Sandling JK, Hom G, Taylor KE, Chung SA, Sun X, et al. A large-scale replication study identifies TNIP1, PRDM1, JAZF1, UHRF1BP1 and IL10 as risk loci for systemic lupus erythematosus. Nat Genet. 2009;41(11):1228-33. Epub 2009/10/20. doi: 10.1038/ng.468. PubMed PMID: 19838195; PubMed Central PMCID: PMCPMC2925843.

29. International Consortium for Systemic Lupus Erythematosus G, Harley JB, Alarcon-Riquelme ME, Criswell LA, Jacob CO, Kimberly RP, et al. Genome-wide association scan in women with systemic lupus erythematosus identifies susceptibility variants in ITGAM, PXK, KIAA1542 and other loci. Nat Genet. 2008;40(2):204-10. Epub 2008/01/22. doi: 10.1038/ng.81. PubMed PMID: 18204446; PubMed Central PMCID: PMCPMC3712260.

30. Wang YF, Zhang Y, Lin Z, Zhang H, Wang TY, Cao Y, et al. Identification of 38 novel loci for systemic lupus erythematosus and genetic heterogeneity between ancestral groups. Nat Commun. 2021;12(1):772. Epub 2021/02/05. doi: 10.1038/s41467-021-21049-y. PubMed PMID: 33536424; PubMed Central PMCID: PMCPMC7858632.

31. Yin X, Kim K, Suetsugu H, Bang SY, Wen L, Koido M, et al. Meta-analysis of 208370 East Asians identifies 113 susceptibility loci for systemic lupus erythematosus. Ann Rheum Dis. 2020. Epub 2020/12/05. doi: 10.1136/annrheumdis-2020-219209. PubMed PMID: 33272962; PubMed Central PMCID: PMCPMC8053352.

32. Lessard CJ, Sajuthi S, Zhao J, Kim K, Ice JA, Li H, et al. Identification of a Systemic Lupus Erythematosus Risk Locus Spanning ATG16L2, FCHSD2, and P2RY2 in Koreans. Arthritis Rheumatol. 2016;68(5):1197-209. Epub 2015/12/15. doi: 10.1002/art.39548. PubMed PMID: 26663301; PubMed Central PMCID: PMCPMC4981330.

33. Martin JE, Assassi S, Diaz-Gallo LM, Broen JC, Simeon CP, Castellvi I, et al. A systemic sclerosis and systemic lupus erythematosus pan-meta-GWAS reveals new shared susceptibility loci. Hum Mol Genet. 2013;22(19):4021-9. Epub 2013/06/07. doi: 10.1093/hmg/ddt248. PubMed PMID: 23740937; PubMed Central PMCID: PMCPMC3766185.

34. Graham RR, Cotsapas C, Davies L, Hackett R, Lessard CJ, Leon JM, et al. Genetic variants near TNFAIP3 on 6q23 are associated with systemic lupus erythematosus. Nat Genet. 2008;40(9):1059-61. Epub 2009/01/24. doi: 10.1038/ng.200. PubMed PMID: 19165918; PubMed Central PMCID: PMCPMC2772171.

35. Wang YF, Wei W, Tangtanatakul P, Zheng L, Lei Y, Lin Z, et al. Identification of Shared and Asian-Specific Loci for Systemic Lupus Erythematosus and Evidence for Roles of Type III Interferon Signaling and Lysosomal Function in the Disease: A Multi-Ancestral Genome-Wide Association Study. Arthritis Rheumatol. 2022;74(5):840-8. Epub 2021/11/17. doi: 10.1002/art.42021. PubMed PMID: 34783190.

36. Alarcon-Riquelme ME, Ziegler JT, Molineros J, Howard TD, Moreno-Estrada A, Sanchez-Rodriguez E, et al. Genome-Wide Association Study in an Amerindian Ancestry Population Reveals Novel Systemic Lupus Erythematosus Risk Loci and the Role of European Admixture. Arthritis Rheumatol. 2016;68(4):932-43. Epub 2015/11/26. doi: 10.1002/art.39504. PubMed PMID: 26606652; PubMed Central PMCID: PMCPMC4829354.

37. Zhang YM, Zhou XJ, Wang YN, Liu XZ, Wang YF, Lau YL, et al. Shared genetic study gives insights into the shared and distinct pathogenic immunity components of IgA nephropathy and SLE. Mol Genet Genomics. 2021;296(4):1017-26. Epub 2021/06/03. doi: 10.1007/s00438-021-01798-7. PubMed PMID: 34076728.

38. Lee YH, Bae SC, Choi SJ, Ji JD, Song GG. Genome-wide pathway analysis of genome-wide association studies on systemic lupus erythematosus and rheumatoid arthritis. Mol Biol Rep. 2012;39(12):10627-35. Epub 2012/10/12. doi: 10.1007/s11033-012-1952-x. PubMed PMID: 23053960.

39. Hu J, Li C, Wang S, Li T, Zhang H. Genetic variants are identified to increase risk of COVID-19 related mortality from UK Biobank data. Hum Genomics. 2021;15(1):10. Epub 2021/02/05. doi: 10.1186/s40246-021-00306-7. PubMed PMID: 33536081; PubMed Central PMCID: PMCPMC7856608.

40. Sun BB, Maranville JC, Peters JE, Stacey D, Staley JR, Blackshaw J, et al. Genomic atlas of the human plasma proteome. Nature. 2018;558(7708):73-9. Epub 2018/06/08. doi: 10.1038/s41586-018-0175-2. PubMed PMID: 29875488; PubMed Central PMCID: PMCPMC6697541.

41. Suhre K, Arnold M, Bhagwat AM, Cotton RJ, Engelke R, Raffler J, et al. Connecting genetic risk to disease end points through the human blood plasma proteome. Nat Commun. 2017;8:14357. Epub 2017/02/28. doi: 10.1038/ncomms14357. PubMed PMID: 28240269; PubMed Central PMCID: PMCPMC5333359.
