# Supplementary material for: COVID-19 and systemic lupus erythematosus genetics: A balance between autoimmune disease risk and protection against infection
Source: PLoS Genet. 2022 Nov 3;18(11):e1010253. doi: 10.1371/journal.pgen.1010253 (PMC9632821; doi:10.1371/journal.pgen.1010253)
Supplement: Attachment — Submitted filename: 06092022_PLOS_reviewer_3.docx [file pgen.1010253.s002.docx]

**We thank the reviewer for a detailed and helpful review. The paper has been re-written to address the points raised. In particular with respect to describing the study, clarification on methodology and presentation of some results. Further details are below in response to each point in turn.**

Reviewer #3:

1- Wang et al. focused on the genetic features of COVID-19 and SLE. However, although the idea is novel and interesting, the author did not provide sufficient justification for why these two traits, especially since recent studies have shown no higher rate of severe COVID-19 in patients with SLE; rather the most severe outcome is due to comorbidities or untreated SLE. (PMID: 35172961) Although a recent survey showed patients with SLE do have a lower serological response to the vaccine, this was identified to be associated with several types of medicinal uses. Another recent study found that most patients with SLE and confirmed COVID-19 were able to produce and maintain a serological response despite the use of a variety of immunosuppressants (PMID: 34075358)

**Response:** **We have re-written the paper to make clear that our study is on the shared genetic risk alleles and observed genetic correlation. We were not working on the hypothesis of comorbidity but on the shared genetics.**

2- Authors used Coloc to examine shared causal SNPs of traits (COVID-19 and SLE). However, this does not establish a causal relationship between the traits. The author could consider using Mendelian randomization to identify biological mediators in the causal pathways using GWAS and pQTL results that are available in the public domain.

**Response:** **Mendelian randomization is an interesting idea but best placed in the context of the causal effect of altered gene and/or protein expression as causal for the two diseases, rather than a causal relationship between SLE and severe COVID-19. As noted in comment-1 we do not believe that either disease is casual of the other but rather that they do share genetic causal alleles, so there is pleiotropy.**

3- Line 30: the COVID GWAS study was conducted as a trans-ethnic study. The controls were matched to the cases for genetic ancestry and other factors. The three SLE GWAS used only European ancestry individuals. The authors should address the population discrepancy in performing a meta-analysis and wehether any of the variants identified have higher frequency in certain populations.
Ref 2: North Americans of European descent
Ref 3: women of European ancestry
Ref 4: mainly southern European ancestry

**Response:** **Yes the COVID-19 hg GWAS was trans-ethnic, while the SLE was European only. Meta-analysis across ancestry have been shown to be powerful in detecting association when causal loci are shared across ancestry, which has been shown to be the case in SLE^1,2^. We agree that it is important that each contributing study has been properly controlled for population structure. We have now included information on genomic inflation in the SLE meta-analyses on lines 441 – 442 and 453 in which we did not find any evidence of inflation.**

4- Line 58: cis-eQTL signals for PDE4A in what cell types? And is this cell type relevant to SLE?

**Response:** **We have clarified the cell types on lines 108 – 109. The cells were collected from left artery tibial, peripheral tibial artery from gastrocnemius region tibial tissues^3^. This tissue type is heterogeneous and the relevance to SLE is unclear, we have now made this clear on line 164 – 165.**

5- Line 78: the authors looked into the functional effects of SNPs at the TYK2 locus. The authors selected a set of 21 IFN-induced genes that are upregulated in SLE in the pQTL dataset published by Zhen et al. Although two proteins, SERPING1 and CXCL10, were both found to have a significant trans-pQTL, the authors did not offer a table with the summary statistics. It’s also unclear why the authors limited the pQTL search only to the subset of the 21 IFN-induced genes, as there may be other interesting potential pathways involved in the response of the risk alleles. This could be an excellent opportunity to decipher the conflicting results seen in signal-A and signal-B.

**Response:** **This is an interesting and helpful point, and it was an oversight in omitting the summary statistics for the pQTLs, these are now in supplementary table 8. We limited the search to these genes as they have been established as altered in SLE, so we did not want to limit our power by exploring a wider set of proteins that may or may not be relevant. We agree that this would be an excellent avenue to explore further with a more expansive set of genes.**

6- Line 84: authors performed a trans-trait meta-analysis. The idea is interesting; however, it did not address the validity of the results. It is well known that mixing dissimilar studies results in reduced effectiveness. COVID and SLE may have overlapping genomic features, but there is no evident similarity in disease etiology. That being said, I do not have high confidence in the findings of CLEAC1A and the analysis presented.

**Response:** **The pathology of SLE includes activation of viral sensing and antiviral immune response. We make this point in the Introduction to rationalize the comparison of the two diseases. We are not the first to perform a cross-trait meta-analysis to identify shared genetic associations. These studies work well due to the extensive pleiotropy in complex disease and comparing traits increases power, see for example ^4,5^.**

1.	Wang, Y.F. *et al.* Identification of 38 novel loci for systemic lupus erythematosus and genetic heterogeneity between ancestral groups. *Nat Commun* **12**, 772 (2021).

2.	Morris, D.L. *et al.* Genome-wide association meta-analysis in Chinese and European individuals identifies ten new loci associated with systemic lupus erythematosus. *Nature Genetics* **48**, 940-+ (2016).

3.	Consortium, G.T. The GTEx Consortium atlas of genetic regulatory effects across human tissues. *Science* **369**, 1318-1330 (2020).

4.	Zhuang, Z., Yao, M., Wong, J.Y.Y., Liu, Z. & Huang, T. Shared genetic etiology and causality between body fat percentage and cardiovascular diseases: a large-scale genome-wide cross-trait analysis. *BMC Med* **19**, 100 (2021).

5.	Li, X. & Zhu, X. Cross-Phenotype Association Analysis Using Summary Statistics from GWAS. *Methods Mol Biol* **1666**, 455-467 (2017).
